# A Scoping Review of Methods, Guidelines, and Protocols Used for Dose Reduction or Discontinuation of Antipsychotic Medication in People Affected by Psychotic Disorders

**DOI:** 10.1093/schbul/sbaf181

**Published:** 2025-10-28

**Authors:** Isabelle Jeffares, Dara Friars, Helena Plapp, Kelly Allott, Cathal Cadogan, Dolores Keating, Catherine Mooney, Karen O’Connor, Brian O’Donoghue

**Affiliations:** Department of Psychiatry, School of Medicine, University College Dublin, Dublin, D04 V1W8, Ireland; Mental Health Services, Royal Melbourne Hospital, Melbourne, VIC 3050, Australia; Cluain Mhuire Community Mental Health Service, Blackrock, Co Dublin, A94 VK83, Ireland; Orygen, Centre for Youth Mental Health, Parkville, VIC 3052, Australia; Centre for Youth Mental Health, The University of Melbourne, Parkville, VIC 3010, Australia; School of Pharmacy and Pharmaceutical Sciences, Trinity College Dublin, College Green, Dublin 2, D02 PN40, Ireland; Pharmacy Department, Saint John of God University Hospital, Dublin, Stillorgan,Dublin, D94 FH92, Ireland; School of Computer Science, University College Dublin, Dublin, D04 B1W8, Ireland; RISE Early Intervention for Psychosis Service, Blackpool, Co Cork, Ireland; Department of Psychiatry, School of Medicine, University College Dublin, Dublin, D04 V1W8, Ireland

**Keywords:** tapering, psychosis, schizophrenia, guidelines, deprescribing

## Abstract

**Background:**

Individuals affected by psychotic disorders often decide to have a trial of dose reduction or discontinuation of their antipsychotic medications; however, there is a lack of evidence-based guidance for this practice. There is limited knowledge on the different methods for dose reduction or discontinuation of antipsychotic medications in which methods might be the safest and most effective. This scoping review aims to identify methods of antipsychotic dose reduction and discontinuation, to develop a comprehensive inventory of tapering strategies.

**Study Design:**

A systematic search was performed in PubMed, EMBASE, PsycINFO, and Cochrane to identify publications involving antipsychotic dose reduction or discontinuation. We included primary studies, systematic reviews, theoretical papers, and guidelines.

**Study Results:**

A total of 9005 unique articles were identified, and full text screening was conducted on 459 of these. Thirty-two articles were included in the final analysis: 28 related to oral antipsychotic medications and 4 to long acting injectables. Three broad categories of dose reduction/discontinuation were identified—immediate, rapid (<4 weeks), and gradual (short 4-26 weeks and long≥26 weeks). Further subtypes were identified, with reduction based on either the original starting dose or the most recent dose after the last reduction. The hyperbolic method estimates the dose reduction based on a gradual reduction in the D_2_ receptor occupancy.

**Conclusions:**

A wide range of antipsychotic tapering methods have been implemented internationally with no consensus on the best approach. The results of this scoping review could help to inform research on establishing the most effective methods of antipsychotic discontinuation.

## Introduction

Schizophrenia and psychotic illnesses affect up to 1% of the population worldwide. ^1^ These conditions frequently co-occur with other metal illnesses^2^ and are associated with poor physical health^3–6^ and higher mortality.^7^^,^^8^ Antipsychotics are first-line treatment for psychotic disorders, to reduce symptoms and prevent relapse.^9^ However, these medications have limited effect on psychosocial function and individuals frequently report a desire to discontinue antipsychotic medications due to debilitating side effects and reduced quality of life.^10^^,^^11^ Some individuals with first-episode psychosis will never experience a further episode,^12–14^ hence continued treatment with antipsychotic medication would unnecessarily expose these individuals to side effects. Therefore, determining the safest method by which to reduce antipsychotic dosage is required.

While several guidance documents support discontinuation of antipsychotic medication,^15^ there is a clear absence of specific recommendations on how (and when) to safely discontinue medication in people recovering from psychosis. Maintenance antipsychotic treatment continues to be the standard approach, without consensus on the appropriate duration of the maintenance phase, for example, from 1 year^16^^,^^17^ to 5 years or indefinitely.^18–21^ Longitudinal cohort studies have found that rates of antipsychotic discontinuation are high, with over 60% of people with a first episode of psychosis discontinuing antipsychotic medications within a median of 6 months^22^ and by 5 years, less than 10% of people were taking antipsychotic medications continuously.^23^ In the large Danish registry study, it was found that after 5 years, 38.6% of individuals were not taking antipsychotic medications, 48.7% had discontinued but then resumed treatment, and 3.4% had sustained discontinuation after the second year.^23^ Several international trials have explored antipsychotic discontinuation (eg, versus continuation) in individuals with psychotic illnesses^24–27^ and a number of systematic reviews have evaluated patient outcomes following discontinuation^28–36^ or antipsychotic switching.^37^ Although a wide range of methods were applied in these studies, little is known about the advantages of one discontinuation strategy over another, and as such, the most effective method is yet to be established.

The global burden of psychotic illness presents significant challenges for treatment and recovery.^38^^,^^39^ Given the potential implications of long-term antipsychotic use, and increased risk of relapse due to medication non-adherence, there is an urgent need for explicit guidelines on discontinuation of antipsychotic medications. To date, no scoping review has been conducted to provide a global picture of the tapering strategies in use internationally. This review addresses this gap by comprehensively mapping the literature to identify antipsychotic dose reduction and discontinuation strategies.

## Methods

### Protocol and Registration

The protocol for this scoping review was registered with OSF Registries.^40^ This study followed the 5-stage methodological framework for scoping reviews set out by Arksey and O’Malley,^41^ later advanced by Levac and colleagues,^42^ and Peters et al.^43^ The findings were reported in line with the Joanna Briggs Institute (JBI) Preferred Reporting Items for Systematic reviews and Meta-Analysis extension for Scoping Reviews (PRISMA-ScR) reporting guidelines.^44^

### Eligibility Criteria

Peer-reviewed and grey literature were systematically searched for eligible papers. This review applied the components of the PCC (population, concept, context) framework in accordance with JBI recommendations^44^:

- **Population:** Individuals with a primary diagnosis of psychotic disorder, including Schizophrenia Spectrum Disorders (eg, Schizophrenia, Schizoaffective disorder, Schizophreniform disorder, Delusional disorder), Affective psychotic disorders (Bipolar disorder, depression with psychosis), or other psychotic disorders (eg, Brief psychotic disorder, Substance-induced psychotic disorder, Psychotic disorder not otherwise specified).
- **Concept:** All methods of antipsychotic discontinuation or dose reduction, including abrupt termination or gradual tapering of antipsychotic medication. All types of antipsychotic medication were eligible (eg, first- and second-generation antipsychotic) and formulations (oral vs long acting injectables).
- **Context:** There were no restrictions regarding study setting or context (inpatient, outpatient, community).

### Information Sources

Four electronic databases (Medline, Embase, PsycINFO (Ovid), and the Cochrane Library) were searched for peer-reviewed papers published in English since 2000, to ensure a focus on contemporary interventions for antipsychotic tapering. Original primary research studies (and protocols) were eligible, including randomized controlled trials (RCTs), non-randomized studies (eg, single group before and after studies, observational cohort studies, case–control designs), and secondary analyses. Clinical guidelines were eligible, in addition to systematic reviews, literature reviews, and theoretical papers if they reported a new method or guideline for antipsychotic discontinuation. A “new method” is defined as a tapering strategy developed by the authors, based on the findings of clinical studies or literature included in the review. Only articles which reported their tapering protocol in sufficient detail were included (ie, information on the dosing intervals, amounts reduced, and duration of tapering). Clinical guidelines were identified through international guideline repositories and the websites of professional organizations (Table S1). A search for relevant grey literature was performed using Google Scholar. A structured citation search was also completed on all systematic reviews that qualified for full text review, and all studies included in the final analysis.

Studies which involved immediate discontinuation of medication (eg, due to adverse side effects) were excluded, unless the authors specified that the aim of the intervention was discontinuation. Likewise, studies that measured discontinuation as an outcome (eg, time to discontinuation) were excluded, as were clinical trials in which the discontinuation arm simply acted as a comparison group. Studies of polypharmacy or antipsychotic switching were not eligible. Case studies, research reports, and book chapters were excluded, in addition to theses and conference proceedings.

### Search

The systematic search strategy was developed with support from an academic librarian and through consultation with literature on antipsychotic tapering (Table S2). We systematically searched MEDLINE, EMBASE, PsycINFO (Ovid), and the Cochrane Library. Searches were performed on August 13, 2024, using subject headings, keywords, and synonyms relating to the population (people with psychosis) and relevant concept terms (antipsychotic terms, discontinuation or dose reduction terms). The search results from each database were imported and de-duplicated in *Endnote 21*.

### Selection of Sources of Evidence

The titles and abstracts of all articles were screened in *Covidence* by 2 independent reviewers, who were briefed on the screening process and provided with a copy of the inclusion and exclusion criteria (Table S3). Reviewers met regularly throughout screening to clarify queries and to ensure consistency. Each record was reviewed by the first author and one of the second reviewers, and disagreements were resolved with a third reviewer until consensus was reached. Where the details of the tapering protocol were unclear, authors were contacted for clarification. The screening process is summarized in Figure 1.

**Figure 1:**
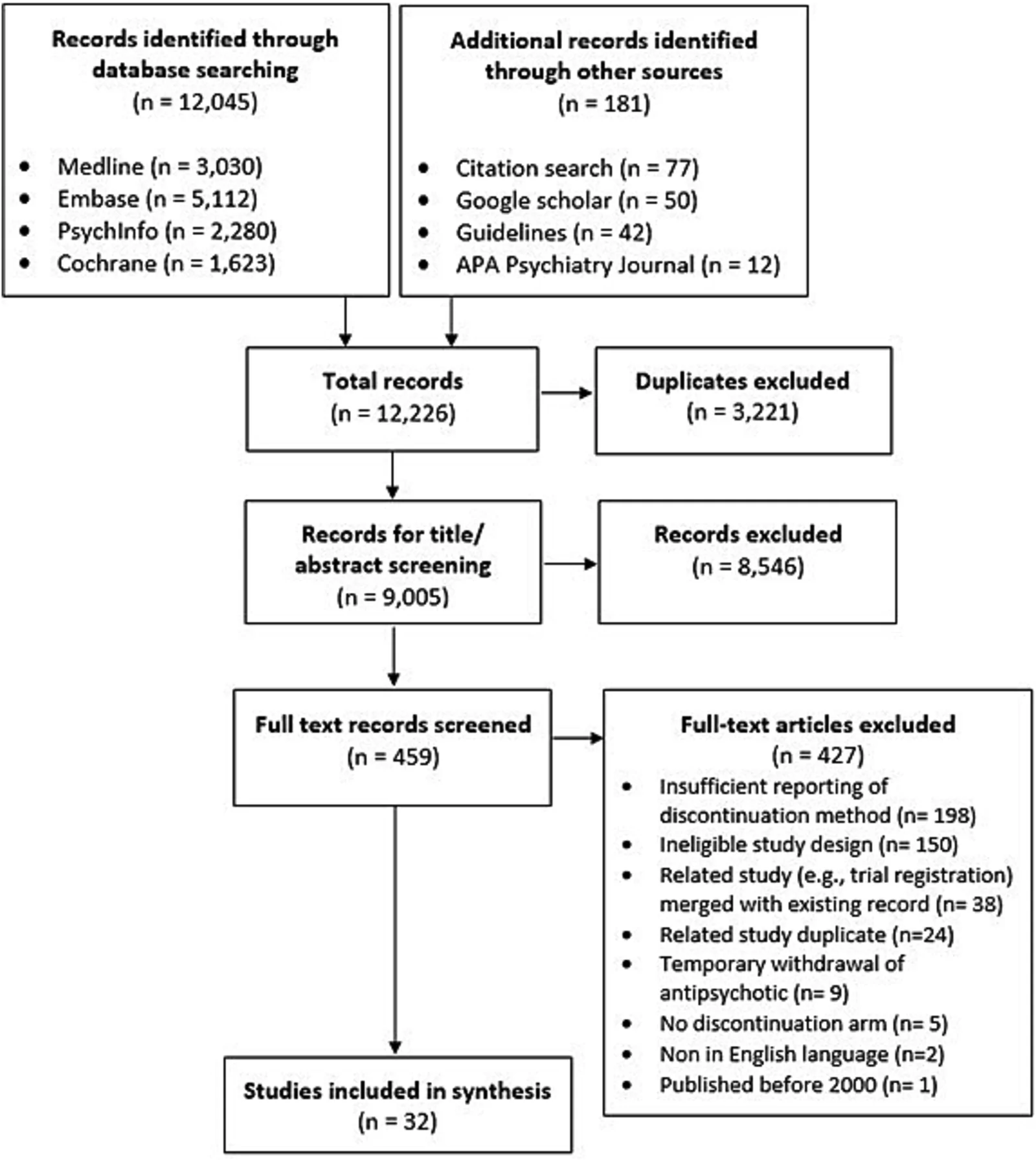
PRISMA Flow Diagram of Eligible Studies

### Data Charting Process

Data were charted and entered into a standard recording form developed in Excel a priori, in line with JBI guidelines.^44^ The data charting form was pilot tested with different types of publication and shaped by the articles included in the final analysis. Data were extracted by one reviewer (IJ) and a second author (BOD) reviewed the extracted data to ensure accuracy and completeness, as per established guidance.^45^ During data extraction, papers relating to the primary study were identified by the search (eg, trial registrations, secondary analysis of trial data, etc.) and merged with the original publication. These papers included additional details about the tapering interventions and are referenced in Table S4.

### Data Items

The following data were extracted for analysis in this review: (1) Study characteristics—study title, author, year of publication, and country of origin; (2) Study methodology and participants—study design, study duration, follow-up duration, diagnosis, episode, duration of illness, sample size, age, gender, primary outcome, and setting; (3) Antipsychotic tapering protocols—medication name, mode of administration (oral/injectable), the duration of tapering, and the tapering schedule (the number, amount, and frequency of medication reductions, eg, 4 dose reductions of 20% each, every 4 weeks).

### Critical Appraisal of Individual Sources of Evidence

A formal appraisal of methodological quality of studies was not undertaken as the aim of a scoping review is to provide a comprehensive overview of available evidence on a subject, regardless of quality.^43^

### Synthesis of Results

Articles were summarized and presented in tables accompanied by narrative description, following validated guidelines for data synthesis.^44^^,^^46^  Table 1 presents the characteristics of included articles, organized by study design. The antipsychotic tapering protocols are grouped by method type and summarized in Table 2, with further detail provided in Table S4. The 4 studies involving tapering of long-acting injectable (LAI) medications are presented at the end of Table 2, separate from studies involving oral preparations or a mixture of both types of medication. Data relating to the tapering methods were synthesized in 3 steps. First, tapering methods were categorized by duration; abrupt methods were defined as tapering over 4 weeks or less, and gradual methods of tapering were defined as those taking place over a longer period. Second, the dose reduction interval was determined and classified as “fixed” or “variable” dose reductions. Third, dose reductions were further differentiated as being taken from the original dose, or the most recent dose of medication. The data were summarized as counts with proportions, averages, and range values.

**Table 1:** Characteristics of Included Studies and Articles (*n* = 32)

**Author, year, country**	**Study design/publication type**	**Study duration (weeks)**	**Follow-up (weeks)**	**Diagnosis**	**Episode**	**Illness duration (years)**	**Sample size (*n*)**	**Mean age in years [range]**	**Gender (% male)**	**Primary outcome (assessment measure)**	**Setting**
Volavka, 2000, USA^48^	RCT	31	16	SSD^a^	n.s.	n.s.	23	40	87	Severity of symptoms (PANSS)	n.s.
Gitlin, 2001, USA^49^	RCT	102	78	SSD^a^	First	2	53	25 [18-45]	87	Relapse	Inpatient
Brown, 2003, USA^50^	RCT	12	12	SSD^a^, affective psychosis	n.s.	n.s.	29	37	66	Drug cravings (CCQ); BPRS18; HRSD17	Outpatient
Kinon, 2004, USA^51^	RCT	35	n.s.	SSD^a^	n.s.	23	95	49 [24-65]	57	Symptoms (TD)	Inpatient
Khazaie and Shakeri, 2005, Iran^52^	RCT	54	54	SSD^a^	n.s.	12	50	34	72	Side effects	Outpatient
Rouillon, 2008, France^53^	RCT	26	26	SCZ	n.s.	n.s.	97	39	68	Relapse	Inpatient
Wang, 2010, China^54^	RCT	78	52	SCZ	n.s.	7	404	33	46	Relapse	Both
Gaebel, 2011, Germany^55^	RCT	112	52	SCZ	First	n.s.	44	33	57	Relapse	Inpatient
Takeuchi, 2013, Japan^56^	RCT	28	28	SCZ	n.s.	14	61	40	61	Cognitive function (RBANS)	Inpatient
Flint, 2019, Canada^57^	RCT^b^	56	36	MDD and psychosis	First	0.5	126	55	38	Relapse	Inpatient
Emsley, 2014, South Africa^58^	RCT^b^	130	104	SSD^a^	First	2-3	33	29	72	Relapse	Both
Zhou, 2018, China^59^	RCT	52	40	SCZ	n.s.	n.s.	75	44	60	Severity of symptoms (PANNS)	Inpatient
Ozawa, 2019, Japan^60^	RCT	52	52	SCZ	n.s.	36	35	64 [45-80]	66	Successful dose reduction	Inpatient
Huhn, 2021, Germany^61^	RCT	26	13	SSD^a^	n.s.	17	20	45	61	Relapse	Outpatient
Stürup, 2022, Denmark^62^	RCT	52	52	SCZ, non-affective disorders	First	2	29	25	41	Remission of symptoms	Outpatient (EIS)
Moncrieff, 2023, UK^24^	RCT	104	78	SSD^a^, non-affective disorders	Both	Up to 20+	253	46 [22-79]	66	Social functioning	Outpatient
Liu, 2023, Taiwan^25^	RCT	104	104	SSD^a^, psychotic disorders	Both	11	96	36	45	Severity of symptoms	Inpatient
Bogers, 2024, The Netherlands^63^	RCT	88	52	SSD^a^	n.s.	2	48	51	58	Neurocognitive function	Inpatient
Weller, 2019, Australia^64^	RCT protocol	104	104	Psychotic disorder, mood disorder	First	N.A.	144 (est.)	N.A.[15-25]	N.A.	Social and Occupational Functioning (SOFAS)	Both (EIS)
Begemann, 2020, The Netherlands^65^	RCT protocol	209	209	SSD^a^, non-affective disorders	First	N.A.	512 (est.)	N.A.[16-60]	N.A.	Personal and social functioning (WHO-DAS)	Both
Rothschild and Duval, 2003, USA^66^	Single group before and after study	69	64	MDD and psychosis	n.s.	4	40	41 [27-64]	40	Depression (HAM-D, BPRS)	Both
Nishikawa, 2007, Japan^67^	Observational cohort study	558	558	SSD^a^	Multi-episode	n.s.	30	33	67	Successful withdrawal	Outpatient
Emsley, 2012, South Africa^66^	Single group before and after study	182	156	SSD^a^	First	n.s.	33	28	58	Symptom recurrence	Outpatient
Graff-Guerrero, 2015, Canada^67^	Single group before and after study	28	26	SSD^a^	n.s.	33	35	60	72	Striatal dopamine D2/3R occupancy	Outpatient
Steingard, 2018, USA^70^	Secondary analysis (controlled before and after study)	287	261	SCZ	n.s.	n.s.	67	47	64	Relapse (hospitalization)	Outpatient
Kim, 2021, Republic of Korea^71^	Single group before and after study	16	12	Psychosis	First	3	39	26 [19-45]	32	Relapse	Inpatient
Horowitz, 2021, UK^11^	Lit review/expert consensus	N/A	N/A	SCZ	Both	N/A	N/A	N/A	N/A	N/A	N/A
Blackman, 2022, UK^72^	Lit review/expert consensus	N/A	N/A	SCZ	n.s.	N/A	N/A	N/A	N/A	N/A	N/A
Hui, 2022, Republic of Korea^15^	Lit review	N/A	N/A	SSD^a^	First	N/A	N/A	N/A	N/A	N/A	N/A
Wunderink, 2024, The Netherlands^73^	Lit review/expert consensus	N/A	N/A	Psychosis	Both	N/A	N/A	N/A	N/A	N/A	N/A
Lehman, 2004, USA^74^	CPG	N/A	N/A	SCZ	First	N/A	N/A	N/A	N/A	N/A	N/A
Grover, 2017, India^75^	CPG	N/A	N/A	SCZ	Both	N/A	N/A	N/A	N/A	N/A	N/A

Abbreviations: AP = antipsychotic; CPG = clinical practice guideline; CCQ = cocaine craving questionnaire; D2/3R = dopamine D2/3 receptor; EIS = early intervention service; est. = estimated; HAM-D = Hamilton Depression Rating Scale; Lit = literature; N/A = not applicable; N.A. = not available; n.s. = not specified; PLCB = placebo; PANNS = positive and negative syndrome scale score; RCT = randomized controlled trial; MDD = major depressive disorder; RBANS = repeatable battery for the assessment of neuropsychological status; SOFAS = social and occupational functioning assessment scale; SCZ = schizophrenia; SSD = schizophrenia spectrum disorder; TD = tardive dyskinesia; WHO-DAS 2.0 disability scale = World Health Organization Disability Assessment Schedule; HRSD17 = 17-item Hamilton Rating Scale for Depression; BPRS18 = 18-item Brief Psychiatric Rating Scale.

a SSD refers to schizophrenia, schizoaffective disorder, and/or schizophreniform disorder.

b Placebo-controlled RCT.

**Table 2:** Description of Dose Reduction and Discontinuation Methods (*n* = 32)

**Author, year**	**Type of intervention**	**Medication to be tapered**	**Med type**	**Time between interval (weeks)**	**Initial dose reduction (% or mg)**	**Reduction from initial or most recent dose**	**Total reductions required**	**Final reduction or dose prior to discontinue**	**Estimated D_2_ receptor occupancy of last dose**	**Total weeks to dose reduction/discontinue**	**Follow-up period (weeks)**	**Method category**
Immediate or rapid discontinuation/dose reduction methods for oral medications (*n* = 8)												
Flint, 2019^57^	D	OLZ	Oral	0	100%	Initial dose	1	n.a.	n.a.	0	36	Immediate
Brown, 2003^50^	D	FGAs ^j^	n.s.	n.s.	n.s.	Initial dose	n.s.	n.a.	n.a.	2-4	12	Rapid—fixed percentage/dose from original dose
Rothschild and Duval, 2003^66^	D	PPZ	Oral	1	25%	Initial dose	4	n.s.	n.s.	4	64	Rapid—fixed percentage/dose from original dose
Kinon, 2004^51^	DR	OLZ	Oral	2	75%	Initial dose	1-2	5 mg or 2.5 mg	37% or 27%	2	n.s.	Rapid—fixed percentage/dose from original dose
Takeuchi, 2013^56^	DR	RIS and OLZ	Oral	4	25%	Initial dose	2	2 mg (RIS)/5 mg (OLZ)	60% (RIS) 37% (OLZ)	4	28	Rapid—fixed percentage/dose from original dose
Graff-Guerrero, 2015^69^	DR	RIS and OLZ	Oral	1	2.5; 0.5 mg	Initial dose	n.s.	1.5 mg (RIS)/7.5 mg OLZ	52.5% (RIS)/52% (OLZ)	2	26	Rapid—fixed percentage/dose from original dose
Ozawa, 2019^60^	DR	RIS and OLZ	n.s.	4	25%	Initial dose	1	n.s.	n.s.	4	52	Rapid—fixed percentage/dose from original dose
Kim, 2021^71^	D	n.s.	n.s.	1	25%	Initial dose	4	n.s.	n.s.	4	12	Rapid—fixed percentage/dose from original dose
Gradual discontinuation/dose reduction methods for oral medications (*n* = 20)												
Rouillon, 2008^53^	DR	OLZ	n.s.	4	2.5 mg	Initial dose	1-4	2.5 mg	27%	≤26	26	Gradual—fixed dose
Gaebel, 2011^55^	DR	RIS and HAL	Oral	1-2	1 mg	n.s.	6-12	1 mg	47% (RIS)	12	52	Gradual—fixed dose
Bogers, 2024^63^	DR	All	Both^c^	n.s.	5 mg	Initial dose	6-24	5 mg HE	82%	6-24	52	Gradual—fixed dose
Lehman, 2004^74^	D	All	n.s.	4	10%	n.s.	10	n.s.	n.s.	43	n.a.	Gradual—fixed percentage from original dose
Wang, 2010^54^	DR	RIS	Oral	1	5%	Initial dose	8	Half of initial dose	-	8	52	Gradual—fixed percentage from original dose
Grover, 2017^75^	DR	All	n.s.	26	20%	Initial dose	5	n.s.	n.s.	130	n.a.	Gradual—fixed percentage from original dose
Steingard, 2018^70^	DR	n.s.	n.s.	13	25-30%	Initial dose	2	n.s.	n.s.	26	261	Gradual—fixed percentage from original dose
Zhou, 2018^59^	DR	RIS and OLZ	n.s.	4	25%	Initial dose	2	2 mg (RIS)/5 mg (OLZ)	60% (RIS)	16	40	Gradual—fixed percentage from original dose
Weller, 2019^64^	Both	All	n.s.	4	25%	Initial dose	3	n.s.	n.s.	13	104	Gradual—fixed percentage from original dose
Huhn, 2021^61^	DR	All (except CLZ)	n.s.	2	16.7%	Initial dose	6	n.s.	n.s.	13	13	Gradual—fixed percentage from original dose
Stürup, 2022^62^	D	See footnote f	Both^c^	4	25%	Initial dose	4	Individual end doses provided	-	26-39	52	Gradual—fixed percentage from original dose
Wunderink, 2024^73^	DR	All	n.s.	FEP: 4 MEP: 13-26	20-30%	Initial dose	4-5	n.s.	n.s.	FEP: 16-20; MEP: 52-104 or 65-130	n.a.	Gradual—fixed percentage from original dose
Volavka, 2000^48^	DR	ALS	Oral	3	50%	Most recent dose	4	n.s.	n.s.	12	16	Gradual—fixed percentage from most recent dose
Nishikawa, 2007^67^	D	SUL	n.s.	26	50%	Most recent dose	4	50 mg (SUL)	-	104	558	Gradual—fixed percentage from most recent dose
Begemann, 2020^65^	D	See footnote g	Oral	2	Variable^**a**^	Combination	12-15	0.25 mg (RIS), 1.25 mg (OLZ). 25 mg (QTP), 2.5 mg (ARP) (see ref 63 for full list)	15% (RIS), 12% (OLZ). 3% (QTP) 78% (ARP)	26-30	209	Gradual—fixed dose
Moncrieff, 2023^24^	Both	All	n.s.	4-8	Variable^**a**^	Initial dose	6-12	2 mg HE	69%	≥52	78	Gradual
Hui, 2022^15^	Both	All	Both^d^	n.s.	≤25%	Most recent dose	n.s.	<1 mg (RIS)	<47% (RIS)	26-52	n.a.	Gradual

*(continued)*

**Table 2:** Continued.

**Author, year**	**Type of intervention**	**Medication to be tapered**	**Med type**	**Time between interval (weeks)**	**Initial dose reduction (% or mg)**	**Reduction from initial or most recent dose**	**Total reductions required**	**Final reduction or dose prior to discontinue**	**Estimated D_2_ receptor occupancy of last dose**	**Total weeks to dose reduction/discontinue**	**Follow-up period (weeks)**	**Method category**
Horowitz, 2021^11^	Both	All	Both^e^	13-26, however smaller reduction could occur every 4 weeks	Based on D_2_ receptor occupancy Initial reduction equivalent to 5-10%	Most recent dose	10	0.05 mg (HAL), 0.10 mg (RIS), 0.40 mg (OLZ), 10 mg (CLZ), 20 mg (QTP), 0.10 mg (AMS)	<10%	Months to years at rate tolerable to individual	n.a.	Gradual—hyperbolic
Blackman, 2022^72^	D	CLZ	Oral	6-12	n.s.	Most recent dose	20 (tailor to patient)	5 mg (CLZ)	2%	≥26	n.a.	Gradual—hyperbolic
Liu, 2023^25^	DR	See footnote h	Both^c^	26	25%	Most recent dose	4^i^	0.5 mg (RIS)/2.5 mg (ARP) every 2^nd^ day	22% (RIS)	≤104	104	Gradual—hyperbolic
Discontinuation/dose reduction methods for LAI antipsychotic medications (*n* = 4)												
Gitlin, 2001^49^	D	FLU-DEC	LAI	0	100%	Initial dose	1	n.a.	n.a.	0 (LAI)	78	Immediate
Khazaie and Shakeri, 2005^52^	DR	FLU-DEC	LAI	6 (interval ↑ by 4 weeks)	66%^b^	Initial dose	9	25 mg FLU-DEC every 6 weeks	n.a.	54	54	Gradual—fixed percentage from original dose
Emsley, 2012^68^	DR	Risperidone	LAI	2	n.s.	n.s.	3	25 mg RIS-LAI	n.a.	≤6	156	Gradual
Emsley, 2014^58^	D	Flupenthixol decanoate	LAI	4	20%^b^	Most recent dose	5	2.5 mg (FLU_DEC)	n.a.	26	104	Gradual

Abrupt dose reduction/discontinuation was defined as tapering over 4 weeks or less; gradual methods of tapering were defined as those taking place over a longer period. Estimated D_2_ receptor occupancy was based on the study by Lako et al.^76^

Abbreviations: AMS = amisulpride; AP = antipsychotic; ARP = aripiprazole; CLZ = clozapine; D = discontinuation; DR = dose reduction; FEP = first episode psychosis; FGA = first generation antipsychotic; FLU_DEC = fluphenazine decanoate; HAL = haloperidol; HE = haloperidol equivalent; LAI = long-acting injectable; Med = medication; MEP = multi-episode psychosis; n.a. = not applicable; n.s. = not specified; PPZ = perphenazine; QTP = quetiapine; SUL = sulpiride.

a The percentage dose reduction was cited as *variable* where a range of antipsychotic medications (at different baseline dosages) were included and reduced.

b Where the percentage dose reduction (for LAI medication) was not cited in the study, an approximate dose reduction was calculated based on available information.

c Both oral and LAI antipsychotic medications were tapered using the same method in three clinical studies; medications were converted to haloperidol/chlorpromazine equivalent dose for comparison.

d For LAIs, the lowest dose should be tried (25 mg for paliperidone palmitate) with regular or prolonged intervals between injections. For monthly aripiprazole injections, patients should be switched to 15 mg of oral aripiprazole and maintained for at least 3-6 months before discontinuation.

e For LAIs, 3-monthly paliperidone may be tapered over 3 years by yearly dose reductions equivalent to approximately 30% D2 occupancy (reducing by 10 percentage points of D2 occupancy every 4 months). Final dosing requires very small doses (eg, 90 mg, equivalent to 30% D2 occupancy). For drugs with short half-lives (eg, clozapine or quetiapine), it may be necessary to reduce doses by 2.5-5 percentage points of D2 occupancy every 6-12 weeks, depending on individual responses.

f Amisulpride, aripiprazole, chlorprothixene, haloperidol, haloperidol decanoate, lurasidone, olanzapine, paliperidone, paliperidone palmitate, perphenazine, perphenazine decanoate, quetiapine, risperidone, sertindole, ziprasidone, zuclopenthixol, zuclopenthixol decanoate.

g Risperidone, olanzapine, quietapine, aripiprazole, haloperidol, zuclopenthixol, sulpride, palperidone, pimozide, lurasidone, clozapine, amisulpride, tiapride, sertindole, chlorprotixene.

h Amisulpride, aripiprazole, clozapine, flupentixol decanoate, haloperidol, lurasidone, olanzapine, paliperidone, quetiapine, risperidone, sulpiride, trifluoperazine, ziprasidone.

i Indefinite reductions possible.

j First Generation Antipsychotics.

Preliminary findings from the scoping review were presented to stakeholders from our research group (including psychiatrists, pharmacists, endocrinologists, people with lived experience of psychosis) and from the *TAPER Consortium,*^47^ a multi-stakeholder group of international experts, including clinicians, researchers, and patient representatives, responsible for making strategic decisions in the field of psychosis research. These experts provided recommendations which informed the final categorization of tapering methods.

## Results

### Selection of Sources of Evidence

In total, 12 226 publications were identified. Following removal of duplicates, 9005 articles went forward for title and abstract screening. A total of 459 records underwent full text review; comprising 382 records from the database searches and an additional 77 publications identified through other sources (clinical guideline repositories, grey literature, and citation searching). Finally, 427 of these records were excluded, leaving 32 publications for inclusion in this scoping review (Figure 1). The most common reasons for exclusion were insufficient reporting of the tapering protocol (*n* = 198; 46%), and ineligible study design (*n* = 150; 35%, eg, case study, conference proceeding, thesis, etc.)

### Characteristics of Sources of Evidence

Thirty-two publications pertain to 26 primary studies^24^^,^^25^^,^^48–71^ and 6 reviews or guidelines^11^^,^^15^^,^^72–75^ were included in this review and are presented in Table 1. Most articles were primary studies (*n* = 26; 81%), including RCTs (*n* = 18) and RCT protocols (*n* = 2), single group before and after studies (*n* = 4), an observational cohort study (*n* = 1), and a secondary analysis (*n =* 1). Antipsychotic maintenance treatment was the most common control group intervention in controlled studies (*n* = 17/21; 81%). The remaining 6 articles comprised 4 literature reviews/consensus papers and 2 clinical practice guidelines describing tapering methods. Articles were identified from 15 countries, including the USA (*n* = 7), the UK (*n* = 3), the Netherlands (*n* = 3), Japan (*n* = 3), China (*n* = 2), South Korea (*n* = 2), South Africa (*n* = 2), Germany (*n* = 2), and Canada (*n* = 2), with the remaining countries representing one study each (Taiwan, France, Denmark, India, Australia, and Iran).

Many articles concerned patients diagnosed with schizophrenia specifically (*n =* 11; 34%), or schizophrenia spectrum disorders (SSD) (SSD includes schizophrenia, schizoaffective disorder, and/or schizophreniform disorder) (*n =* 11; 34%). The remaining papers focused on psychosis or affective psychotic disorders (eg, psychotic depression), either alone (*n =* 5; 16%) or in combination with SDD (*n* = 5; 16%). Where reported (*n =* 16), a larger proportion of articles (*n =* 11; 69%) concerned patients with first-episode psychosis, while 1 study focused on multi-episode psychosis and 5 articles involved both presentations.

#### Characteristics of Primary Studies and Participants

The following section provides an overview of the 26 studies included in this review (Table 1). Participants were recruited from psychiatric hospitals and medical centers, including inpatient settings (*n* = 11), outpatient departments (*n* = 9), or both (*n* = 5). Most studies (*n* = 21/24; 88%) recruited fewer than 100 participants and sample sizes ranged from 20 to 404 individuals. The average age of participants ranged from 25 to 64 years, with higher representation of male patients across studies (*n* = 18/24; 75%). Duration of illness was reported in 16 studies and ranged from 0.5 to 36 years; half of these studies involved patients with enduring psychotic illness of more than 10 years. Psychotic symptoms and relapse were the most commonly measured primary outcomes (*n* = 16; 62%), while functioning in the cognitive, social, and personal dimensions were less frequently measured (*n* = 5). Other primary outcomes included successful withdrawal (*n* = 2), striatal dopamine occupancy (*n* = 1), depression (*n =* 1), and cravings for illicit drugs in individuals with substance dependence (*n* = 1). Most studies were at least 1 year in duration (*n* = 16/26; 62%), and varied in length from 12 weeks to 287 weeks, with the longest study^67^ following patients for more than 10 years (558 weeks). Studies of gradual antipsychotic tapering tended to report longer average follow-up of patients (*M* = 86 weeks), compared with abrupt tapering (*M* = 40 weeks).

### Results of Individual Sources of Evidence

Table 2 presents the antipsychotic tapering methods identified in the 32 included articles. The majority involved dose reduction (*n =* 17; 53%), or dose reduction with a view to discontinuation for eligible patients (*n =* 4; 13%); the remaining 11 articles (34%) focused on discontinuation specifically. Of 26 clinical studies, 15 involved dose reduction, 9 concerned discontinuation, and 2 covered both approaches. A wide range of medications were tapered, including first-generation antipsychotics (*n =* 7; 24%), second-generation antipsychotics (*n =* 10; 35%), or a combination of both (*n =* 12; 41%). Of 19 articles, 10 (52%) concerned oral antipsychotic medications, 4 (22%) focused on LAI medications, and 5 publications (26%) involved both medication types. Tapering of first-generation antipsychotics was more common in older studies (pre-2011) and studies involving LAI medications. Clozapine, an atypical second-generation antipsychotic medication used for treatment-resistant schizophrenia, was tapered alongside other antipsychotic medications in eight articles, and exclusively in 1 article^72^ (*n =* 9; 31%).

#### Description of Tapering Protocols

The majority of articles (*n =* 23; 72%) employed *gradual* antipsychotic dose reduction or discontinuation defined as longer than 4 weeks (3 involving LAIs), while fewer papers (*n =* 9; 28%) involved tapering over 4 weeks or less. Of these, 2 articles employed immediate discontinuation with no steps or dose reduction (1 involving LAI) and all other 7 articles did a dose reduction or discontinuation within 4 weeks. Clinical practice guidelines (*n =* 2) recommended longer tapering of antipsychotics, for example, over months to years, with one recommending tapering of up to 130 weeks.^75^ Seven (21.9%) articles used a rapid dose reduction strategy, and all of these reduced by a fixed percentage or dose from the original dose. The majority of studies utilized a gradual dose reduction.

The amount and frequency of dose reductions were not uniform across articles and varied depending on the tapering method employed. Reductions from the initial (baseline) antipsychotic dose were most common (*n =* 21/29; 73%), followed by reductions from the most recent dose (*n =* 7; 24%) or a combination of these (*n =* 1; 3%). Initial dose reductions ranged from 5%-100%, with reductions of 20%-30% of the baseline dose being the most common (*n =* 13/19; 68%). The duration between dose reductions ranged from 0 to 26 weeks, with most articles (*n =* 22/29; 76%) reporting intervals of 4 weeks or less between reductions. The number of dose reductions varied widely, from 1 to 20 reductions. The hyperbolic method was recommended in two literature reviews/expert consensus papers^11^^,^^72^ and specifically employed in one clinical study,^25^ where four dose reductions were made over a two-year period. Although the authors of this RCT indicate that indefinite dose reductions are possible with the hyperbolic method, this may not translate from theory to practice. Follow-up periods after discontinuation or dose reduction ranged from 12 weeks to 10 years and these are presented in Table 2.

### Synthesis of Results

Following data synthesis, 6 categories of tapering protocol were developed (Figure 2). The initial determinant of classification was based on the timeline of dose reduction or discontinuation and is defined as either: (1) immediate; (2) rapid, or (3) gradual. Immediate was defined as no further medication given once discontinuation was attempted, that is, the medication was just stopped. Rapid was defined as the target dose reduction or discontinuation occurring within 4 weeks and gradual was defined as any method longer than four weeks. Within gradual, the duration of the tapering method can be classified as short (4-25 weeks) or long (≥26 weeks/6 months).

**Figure 2:**
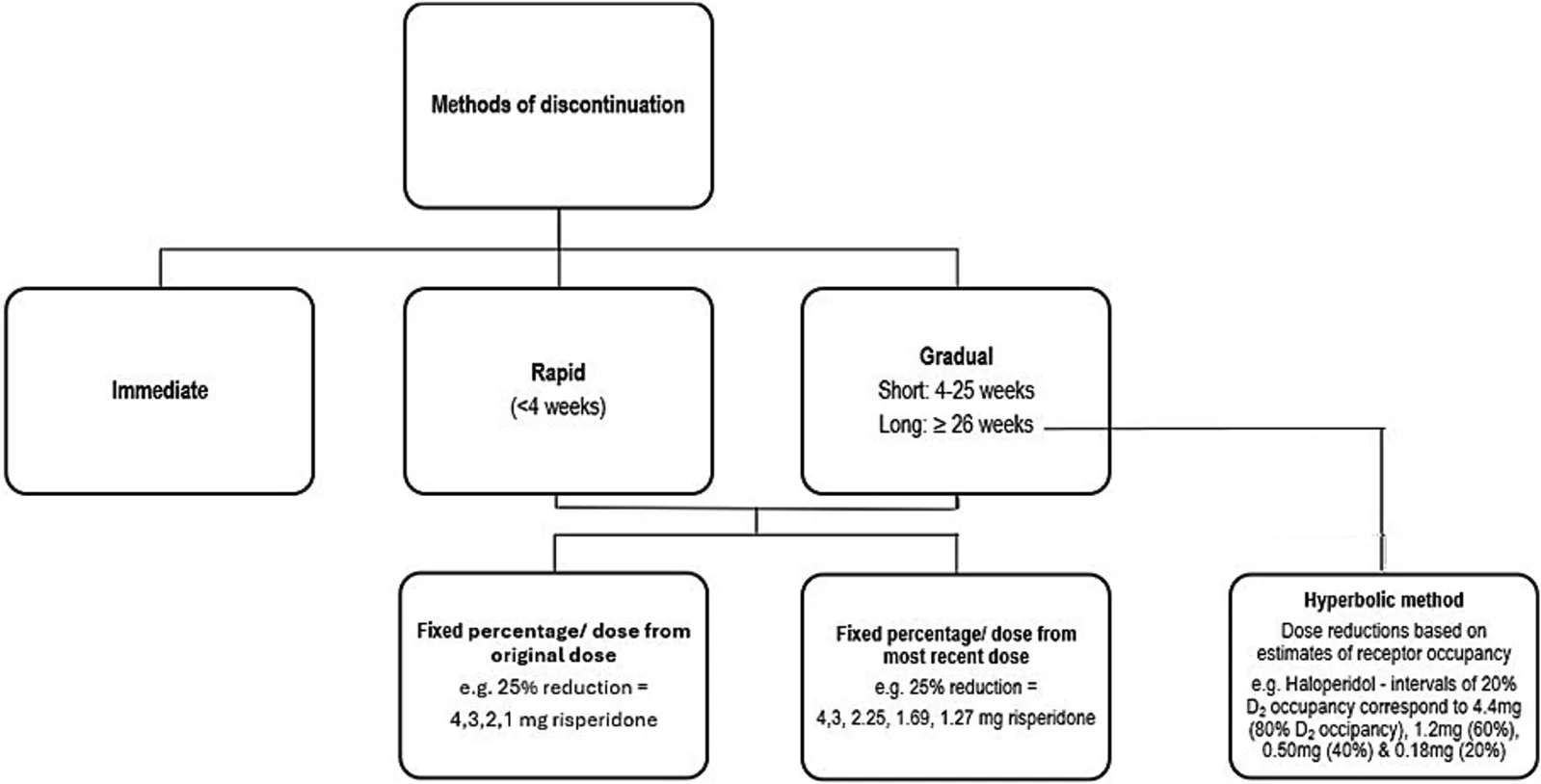
Antipsychotic Dose Reduction and Discontinuation Categories

The second determinant of the classification system of methods is based on the method of determining the increments that the dosage of antipsychotic medication will be reduced. For both rapid and gradual methods, the dose can be reduced by either a fixed percentage or dose from the original starting dose at the time of dose reduction or discontinuation, for example, a 25% reduction from the original starting dose for risperidone would be 4 mg-3 mg-2 mg-1 mg and then stop. Alternatively, the dose reduction or discontinuation could be based on the most recent dose and with this method, a 25% reduction in the above case would be 4 mg-3 mg-2.25 mg-1.69 mg-1.27 mg-0.95 mg and so on. The hyperbolic method was also classified as method of dose reduction or discontinuation that was variable and gradual. In this method, the increment of dose reduction is estimated to reflect the changes in dopamine receptor occupancy that would occur with that change in dose. Therefore, it is aimed to reduce D2 receptor blockage evenly, but this results in variable dose reductions throughout the regime, with very small reductions in the later stages. In the hyperbolic method, dose reductions of between 25% and 50% of the most recent dose are recommended and this would correspond to approximately a 5-10 percentage point decrements of D_2_ occupancy. These reductions are recommended every 3-6 months, however smaller reductions, such as 10% of the most recent dose, could be made every month, as this could make it more tolerable for the individual. For example, with haloperidol a dose of 10 mg will result in an estimated 86% D2 occupancy, at 4.4 mg there will be 80% occupancy, at 1.2 mg there will be 60% occupancy and at 0.50 mg there will be 40% occupancy. The methods highlights that even at a very low dose of 0.25 mg haloperidol, there is approximately 25% occupancy and cessation at this dose would be result in a large drop in occupancy.

Therefore, this results in three broad methods of discontinuation with subtypes.

(1) Immediate—defined as stopping the medication without any reduction
(2) Rapid—within 4 weeks. Subtypes:
  (a) reduction based on fixed percentage/dose from original dose
  (b) reduction based on fixed percentage/dose from most recent dose
(3) Gradual—Short: (4-25 weeks)/Long (≥26 weeks) Subtypes:
  (a) reduction based on fixed percentage/dose from original dose
  (b) reduction based on fixed percentage/dose from most recent dose
  (c) Hyperbolic method (only applicable to the Long Gradual method)

The delineation of 4 weeks classifying a tapering methods as rapid or gradual is an arbitrary cut-off and this is just a first step in attempting to classify methods of dose reduction/discontinuation. We want to emphasize that we are not recommending certain strategies and nor are we recommending that any tapering methods longer than 4 weeks is deemed safe. It should be noted that the majority of studies within the gradual discontinuation category involved tapering over 6 months and hence this led to the proposal to have 2 categories of gradual, namely “short” and “long” with a period of tapering of 6 months differentiating the 2 groups. It should also be noted that the short gradual does not apply to the hyperbolic method, as in this method, each step is recommended to occur at 3-6 month intervals.

## Discussion

This scoping review provides an overview of tapering protocols for dose reduction and discontinuation of antipsychotic medication in psychotic disorders. Dose reduction methods were more common than complete discontinuation, and gradual tapering was employed twice as frequently as abrupt reduction protocols. Combining the results from 32 publications, tapering methods were classified into three broad categories, specifically immediate, rapid (less than 4 weeks), and gradual (short—4-25 weeks and long ≥26 weeks). Within the rapid and gradual methods, there were 2 further subtypes, specifically that the dose could be reduced according to the original or most recent doses. Additionally, the hyperbolic method was categorized as a long gradual method of tapering based on estimated receptor occupancy. The results highlight substantial variation in the tapering approaches being applied internationally and there is a clear need for operationalization of these tapering methods, particularly in terms of the time taken to discontinue medication and the magnitude of dose reductions at each interval.^77^^,^^78^

### Clinical Implications of Findings

Despite a sizable body of literature on this topic, only 2 clinical guidelines were eligible for inclusion in this review,^74^^,^^75^ both of which provided different recommendations; the first proposed gradual tapering of approximately 10% per month until complete antipsychotic discontinuation in patients with first-episode psychosis; the second guideline referred to patients with one or more episodes of psychosis and suggested tapering at a rate of 20% every 6 months until a minimum effective dose is reached. Interestingly, both guidelines do not state whether these reductions are from the original or more recent dose, highlighting that more detail is required when making recommendations around dose reduction/discontinuation. While dose reduction is often part of the process of discontinuation, it is different from complete discontinuation and may account for the variation in guideline documents. Taken together, the results indicate a clear absence of deprescribing information; while several guideline documents advise on antipsychotic maintenance treatment, relatively few consider dose reduction or discontinuation^15^—a limitation of many pharmacological guidelines.^79^

The results of this scoping review suggest a move toward gradual and hyperbolic methods since 2020.^11^^,^^15^^,^^24^^,^^25^^,^^65^^,^^72^ Gradual tapering of antipsychotic medication may reduce withdrawal symptoms and withdrawal psychosis,^70^^,^^80^ hence slower tapering is particularly important for patients who may be at higher risk of relapse on long-term antipsychotic treatment.^81^ Although continued antipsychotic treatment is associated with fewer relapses compared to discontinuation,^36^^,^^82^ studies involving gradual tapering and longer patient follow-up have reported comparable relapse rates between maintenance and discontinuation groups.^27^^,^^70^^,^^83^ In fact, a recent meta-analysis of RCTs reported relapse rates of 77% in patients who discontinued medication immediately, whereas tapering over 1-2 weeks reduced relapse by 20%.^28^ Furthermore, when discontinuation was gradual (over more than 10 weeks), relapse rates dropped to 31%.^28^ Dose reduction is often a lengthy process which demands commitment from patients. Intensive psychoeducation for early signs of relapse when tapering or discontinuing antipsychotics is a vital component of treatment.^81^^,^^84^

In this review, older publications tended to involve first-generation antipsychotics and LAI medications, while second-generation antipsychotics (particularly risperidone and olanzapine) were more common in recent papers. Many studies tapered several medications, including clozapine; a drug prescribed to patients who have not responded to two or more antipsychotic medications. Patients prescribed clozapine tend to have more severe illness and a history of multiple relapses,^72^ hence discontinuation of this medication may require a different approach compared to other drugs. The discontinuation of clozapine is more often due to adverse effects as opposed to having a trial of dose reduction after achieving remission, however it still requires a slow reduction, especially as there is the potential for severe and unpleasant cholinergic discontinuation symptoms (“cholinergic rebound”) if clozapine is stopped too quickly.^85^

There is also limited guidance on how to successfully discontinue LAI antipsychotic medications. It could be assumed that due to the long half-life of these medications, that they would be easier to gradually discontinue. However, some preliminary studies have indicated that abrupt or a gradual reduction of long acting injectables does not lead to a gradual reduction in D_2_ receptor occupancy^86^ and that switching over to low dose oral antipsychotic medication may be required to prevent too rapid a reduction in D_2_ occupancy.^87^

Relapse of psychosis and severity of symptoms were the most common primary outcomes measured, particularly in studies of abrupt antipsychotic tapering, which is associated with higher risk of withdrawal symptoms and relapse.^28^^,^^33^ For patients with psychosis, improved quality of life and capacity to function in social, cognitive, and vocational domains is highly valued.^88–90^ Indeed, some individuals with psychosis would attempt reduction of medication to improve their quality of life, regardless of the risk relapse.^27^^,^^91^^,^^92^ Interestingly, none of the studies in this review measured physical outcomes (eg, weight, metabolic factors) or quality of life as primary outcomes.

Successful discontinuation is related to a number patient-related factors, including relapse history, medication induced dopaminergic hypersensitivity, duration of untreated psychosis, comorbid chronic illnesses, and the co-occurrence of substance use disorder.^93^ It is noteworthy that many of the studies in this review failed to provide sufficiently detailed descriptions of the patient population or the tapering interventions. Data on the duration of illness and psychotic episode (first or multiple) was frequently missing, as well as the length of treatment for the current episode. Age range was less frequently reported and is an important variable for consideration in tapering trials; older patients may respond differently to medications during different phases of their illness, and they are also more likely to suffer from other chronic illnesses, further complicating treatment.^93^

### Reporting of Discontinuation/Dose Reduction Methods

Inadequate reporting of tapering interventions was also evident; many authors did not describe the time between dose reductions, the percentage (or amount) of initial dose reduction, and the number of reductions. This was the commonest reason for studies to have been determined to be ineligible for inclusion, nearly 200 studies excluded on this basis. This emphasizes that the majority of studies on discontinuation/dose reduction are not adequately reporting their methods of tapering. More comprehensive and transparent reporting of interventions is essential, particularly given the high levels of heterogeneity between studies. In a review of antidepressant tapering interventions, the majority of RCTs failed to describe tapering methods in enough detail, preventing further replication and transfer into clinical practice.^94^ Application of the TIDieR checklist would ensure better reporting of intervention components in future studies,^95^^,^^96^ and a modified version could be developed specifically for documenting the components of tapering interventions.

### Reporting Recommendations

The results of this scoping review highlight several potential challenges in establishing the most effective tapering method; first, heterogeneity with regard to tapering interventions makes it difficult to compare methods and to identify the most effective method(s) of tapering; second, the lack of detailed reporting of patient characteristics (eg, length of illness, number of relapses, treatment history) makes it difficult to establish which factors are predictive of successful discontinuation; third, the use of a diverse range of outcome assessments indicates the need for a standardized core outcome set in the field of psychotic illness, to permit greater consistency in the outcome measures used in discontinuation trials and other research studies^97^; fourth, longer follow-up would allow for more accurate measurement of function, particularly in the cognitive, social, and occupational domains.

Finally, tapering protocols will probably need to be tailored to both the medication type and the individual, hence several tapering guidelines may be required. In the HAMLETT RCT, a dose reduction/discontinuation schedule was developed for each individual antipsychotic medication.^65^ In relation to the medication factors, the formulation (oral or intramuscular), D_2_ receptor affinity,^98^ half-life and doses available could all impact on the practicalities of tailoring a discontinuation schedule. Relapses can occur immediately upon dose reduction or discontinuation or in the months and years following discontinuation. While not yet investigated, it is plausible that the pharmacokinetics of specific antipsychotic medications, such as the half-life, could impact upon the more proximal relapses and could be incorporated into the tapering strategy.

In addition, dose reduction and discontinuation strategies should be based on a biologically plausible rationale for what would be the safest and most effective method of discontinuation and not simply on what is the most convenient method based on available formations of medications. For example, tapering methods can often reflect the doses that are available to clinicians to prescribe but the reverse should occur, in that formulations of lower doses should be made available to facilitate more precise prescribing and dispensing.

As previously stated, a large number of studies were excluded from this review as they did not adequately describe the tapering methods employed in their studies. We therefore also recommend that a minimum level of information should be presented for all studies on antipsychotic dose reduction/discontinuation and this should include: (1) the dosing or percentage reductions between intervals and whether it is based on the original or more recent dose; (2) time between intervals; (3) the last dose prior to discontinuation; (4) number of reductions to total discontinuation; and (5) the period of observation or follow-up after discontinuation.

## Strengths and Limitations

This systematic scoping review involved a comprehensive search of peer-reviewed research, grey literature, and clinical practice guidelines, following the PRISMA-ScR guidelines.^46^ To our knowledge, this is the first scoping review to identify and classify antipsychotic tapering strategies applied in research and clinical practice internationally. A strength of this review was the strict inclusion of studies in which the primary aim was to evaluate the effect of antipsychotic dose reduction or discontinuation on participant outcomes. This permitted classification of comparable tapering methods, excluding clinical trials of immediate discontinuation unless the aim of the study aligned with our review.^49^^,^^57^ This review forms part of a larger body of research, which aims to develop guidelines and a decision-making tool for reduction or discontinuation of antipsychotic medications in people recovering from psychosis. It is anticipated that this scoping review will lay the foundation for a second study—a meta-analysis of patient outcomes following discontinuation of antipsychotic medications—to which the results can be applied to identify the most effective and favorable method(s) of antipsychotic discontinuation.

This review has some limitations. High levels of heterogeneity between studies with regard to tapering interventions (duration, dosing protocol, length of follow-up, etc.) and participants (duration of illness, age), makes it difficult to categorize tapering strategies succinctly. A formal assessment of methodological quality of studies was not undertaken; however, many of the studies included in this review were small and underpowered, hence no recommendations for practice can be made in the absence of a quality appraisal. Finally, although there is a substantial literature base on antipsychotic discontinuation, there are considerable deficits with regard to reporting of tapering interventions. Many authors did not describe the tapering strategies in sufficient detail, leading to the exclusion of several studies.

## Conclusions

This review identified a wide range of antipsychotic dose reduction and discontinuation strategies and highlighted considerable variation in tapering approaches; interventions varied widely in terms of their duration, intensity, and dosing protocols, making comparison of tapering strategies a major challenge. High heterogeneity between studies makes it difficult to define the components of the ideal tapering intervention and suggests that a “one size fits all” tapering protocol is unlikely to be feasible. More specific information on deprescribing is needed, most notably the recommended duration of tapering and magnitude of dose reductions. Tailored recommendations for patients with first-episode or multi-episode psychosis is also necessary. Larger in-depth research studies are needed to establish the best approach to tapering antipsychotic medication, such that these interventions can be rigorously tested and comparisons made between studies. Given the significant human, financial, and societal costs attributable to psychotic disorders, developing clinical guidelines for discontinuation of antipsychotic medications is a key health priority.

## Supplementary Material

Supplementary_Table_1_Sources_searched_for_clinical_guidelines_sbaf181

Supplementary_Table_2_Search_strategy_databases_sbaf181

Supplementary_Table_3_Inclusion_and_exclusion_criteria_sbaf181

Supplementary_Table_4_sbaf181

Supplementary_Table_5_PRISMA_ScR_Checklist_sbaf181
