# Supplementary material for: A Scoping Review of Methods, Guidelines, and Protocols Used for Dose Reduction or Discontinuation of Antipsychotic Medication in People Affected by Psychotic Disorders
Source: Schizophr Bull. 2025 Oct 28;52(5):sbaf181. doi: 10.1093/schbul/sbaf181 (PMC13645659; doi:10.1093/schbul/sbaf181)
Supplement: Supplementary file 1 [file supplementary_table_1_sources_searched_for_clinical_guidelines_sbaf181.docx]

Supplementary Table 1. Sources searched for Clinical Practice Guidelines

**Guideline repositories**

Australian Government Clinical Practice Guidelines Portal

Australian National Health and Medical Research Council

British Columbia Ministry of Health

Canadian Medical Association Infobase

German National Disease Management Guideline Programme

Guideline Central

Guidelines International Network

MAGIC repository (Evidence Ecosystem Foundation)

National Guidelines Clearing House

National Institute for Health and Care Excellence (NICE)

New Zealand Guidelines Group

Scottish Intercollegiate Guidelines Network (SIGN)

**Specialist association websites**

American Psychiatric Association

Australia and New Zealand Psychiatric Association

British Association for Psychopharmacology (BAP)

Canadian agency for Drugs and Technology in Health

Canadian Psychiatric Association

Early Psychosis Prevention and Intervention Centre

European Psychiatric Association (EPA)

International Early Psychosis Association (IEPA)

International Psychopharmacology Algorithm Project

Lambeth Early Onset Services, Early Detection and Treatment of Psychosis (TIPS) Norway

National Health Service (NHS) Trust

ORYGEN Australia, the National Centre of Excellence in Youth Mental Health

Prevention and Early Intervention for Psychosis Programme Canada

South London and Maudsley NHS Trust Prescribing Guidelines

Substance Abuse and Mental Health Services Administration

Texas Medication Algorithm Project, World Psychiatric Association

Veterans Affairs United States

World Psychiatric Association

World Society of Biological Psychiatry
