# Supplementary material for: A Scoping Review of Methods, Guidelines, and Protocols Used for Dose Reduction or Discontinuation of Antipsychotic Medication in People Affected by Psychotic Disorders
Source: Schizophr Bull. 2025 Oct 28;52(5):sbaf181. doi: 10.1093/schbul/sbaf181 (PMC13645659; doi:10.1093/schbul/sbaf181)
Supplement: Supplementary file 2 [file supplementary_table_2_search_strategy_databases_sbaf181.docx]

Supplementary Table 2. Database search strategy

| **PubMed (MEDLINE)** | | **Number of records** |
| --- | --- | --- |
| 1 | Psychotic Disorders[MeSH:NoExp] OR Affective Disorders, Psychotic[MeSH] OR Schizophrenia[MeSH] OR Delusions[MeSH] | 163,070 |
| 2 | delusion*[Title/Abstract] OR psychosis[Title/Abstract] OR psychoses[Title/Abstract] OR psychotic[Title/Abstract] OR schizo*[Title/Abstract] | 228,927 |
| 3 | #1 OR #2 | 258,158 |
| 4 | Antipsychotic Agents[MeSH] OR antipsychotic agents[Pharmacological Action] OR Psychotropic Drugs[Mesh:NoExp] | 152,948 |
| 5 | antipsychotic*[Title/Abstract] OR anti-psychotic*[Title/Abstract] OR neuroleptic*[Title/Abstract] | 67,035 |
| 6 | #4 OR #5 | 175,362 |
| 7 | Drug Tapering[MeSH] | 492 |
| 8 | discontinu*[Title/Abstract] OR dose reduc*[Title/Abstract] OR taper*[Title/Abstract] OR withdr*[Title/Abstract] OR ceas*[Title/Abstract] OR cessation[Title/Abstract] NOT smoking[Title/Abstract] | 417,230 |
| 9 | #7 OR #8 | 417,310 |
| 10 | #6 AND #9 | 9,434 |
| 11 | #10 AND #3 | 4,388 |
| 12 | #11 AND Limit to English | 4,033 |
| 13 | #11 AND Limit to English AND year 2000-2024 | 3,030 |
| **EMBASE** | | **Number of records** |
| 1 | 'psychosis'/exp OR 'schizophrenia spectrum disorder'/exp OR 'delusion'/exp | 370,198 |
| 2 | 'delusion*':ti,ab OR 'psychosis':ti,ab OR 'psychoses':ti,ab OR 'psychotic':ti,ab OR 'schizo*':ti,ab | 306,956 |
| 3 | #1 OR #2 | 428,829 |
| 4 | 'neuroleptic agent'/exp OR 'psychotropic agent'/de | 352,962 |
| 5 | 'antipsychotic*':ti,ab OR 'anti-psychotic*':ti,ab OR 'neuroleptic*':ti,ab | 99,644 |
| 6 | #4 OR #5 | 367,909 |
| 7 | 'drug dose reduction'/exp | 101,036 |
| 8 | ('discontinu*':ti,ab OR 'dose reduc*':ti,ab OR 'taper*':ti,ab OR 'withdr*':ti,ab OR 'ceas*':ti,ab OR 'cessation':ti,ab) NOT 'smoking':ti,ab | 641,154 |
| 9 | #7 OR #8 | 717,108 |
| 10 | #6 AND #9 | 24,787 |
| 11 | #10 AND #3 | 11,893 |
| 12 | #11 AND Limit to English | 11,219 |
| 13 | #11 AND Limit to English AND year 2000-2024 | 9,910 |
| 14 | #13 AND EMBASE only | 5,112 |
| **PsychInfo** | | **Number of records** |
| 1 | DE "Psychosis" OR DE "Affective Psychosis" OR DE "Delusional Disorder" OR DE "Paranoid Psychosis" OR DE "Schizophrenia" | 134,516 |
| 2 | TI (delusion* OR psychosis OR psychoses OR psychotic OR schizo*) OR AB (delusion* OR psychosis OR psychoses OR psychotic OR schizo*) | 197,513 |
| 3 | #1 OR #2 | 204,972 |
| 4 | DE "Neuroleptic Drugs" OR MM "Psychotropic Drugs" | 24,328 |
| 5 | TI (antipsychotic* OR anti-psychotic* OR neuroleptic*) OR AB (antipsychotic* OR anti-psychotic* OR neuroleptic*) | 43,557 |
| 6 | #4 OR #5 | 46,927 |
|  | *-no suitable Mesh terms-* |  |
| 7 | TI (discontinu* OR dose reduc* OR taper* OR withdr* OR ceas* OR cessation) NOT TI (smoking) OR AB (discontinu* OR dose reduc* OR taper* OR withdr* OR ceas* OR cessation) NOT AB (smoking) | 86,418 |
| 8 | #6 AND #7 | 4986 |
| 9 | #8 AND #3 | 3,284 |
| 10 | #9 AND Limit to English | 3,100 |
| 11 | #9 AND Limit to English AND year 2000-2024 | 2,280 |
| **Cochrane CENTRAL** | | **Number of records** |
| 1 | Schizophrenia Spectrum and Other Psychotic Disorders (exp) OR Delusions (exp) | 12,357 |
| 2 | (delusion* OR psychosis OR psychoses OR psychotic OR schizo*):ti,ab | 25,977 |
| 3 | #1 OR #2 | 27,333 |
| 4 | Antipsychotic Agents (exp) OR Psychotropic Drugs[**No** exp, single Mesh Term] | 7,023 |
| 5 | (antipsychotic* OR anti-psychotic* OR neuroleptic*):ti,ab | 10,494 |
| 6 | #4 OR #5 | 13,721 |
| 7 | Drug Tapering (exp) | 55 |
| 8 | (discontinu* OR dose reduc* OR taper* OR withdr* OR ceas* OR cessation):ti,ab NOT(smoking):ti,ab | 175,603 |
| 9 | #7 OR #8 | 175,608 |
| 10 | #6 AND #9 | 2,758 |
| 11 | #10 AND #3 | 1,958 |
| 12 | #11 AND Limit to English | 1,902 |
| 13 | #11 AND Limit to English AND year 2000-2024 | 1,623 |
