# Supplementary material for: A Scoping Review of Methods, Guidelines, and Protocols Used for Dose Reduction or Discontinuation of Antipsychotic Medication in People Affected by Psychotic Disorders
Source: Schizophr Bull. 2025 Oct 28;52(5):sbaf181. doi: 10.1093/schbul/sbaf181 (PMC13645659; doi:10.1093/schbul/sbaf181)
Supplement: Supplementary file 3 [file supplementary_table_3_inclusion_and_exclusion_criteria_sbaf181.docx]

Supplementary table 3. Inclusion and exclusion criteria for screening

|  | **Inclusion criteria** | **Exclusion criteria** |
| --- | --- | --- |
| **Population** | - Individuals with a primary diagnosis of any psychotic disorder are eligible (based on DSM/ICD criteria, clinical diagnosis, register-based diagnosis, or structured interview). - Other mental disorders presenting with psychosis, e.g., bipolar disorder with mania, or depression with psychosis. - Any episode of psychosis. - Any age. | - Non-psychotic mental illness (e.g., anxiety, depression) or organic disorders (e.g., brain trauma, stroke, Parkinson’s, Alzheimer’s Disease, Dementia). |
| **Concept** | - Included articles must describe the method of discontinuation or dose reduction in sufficient detail (i.e. information on the dosing intervals, amounts reduced, and duration of tapering). - All methods of antipsychotic discontinuation or dose reduction are acceptable (e.g., abrupt termination or gradual reduction). - Studies involving discontinuation of an antipsychotic while the patient remains on a mood stabiliser (e.g., lithium) or an antidepressant are eligible. | - Studies which do not describe the discontinuation method in sufficient detail. - Studies of polypharmacy or antipsychotic switching, which replace one antipsychotic medication with another (unless an “uncontaminated” discontinuation group is included in the study). |
| **Context** | - Studies delivered in any country or setting are eligible, e.g., hospitals, inpatient settings, outpatient departments, and the community. | - Not applicable. |
| **Study design/ publication types** | - *Primary studies:* - Randomised controlled trials - Non-randomised controlled trials - Single group before and after studies - Observational cohort studies - Longitudinal studies - Case-control designs - Qualitative studies - Study protocols - Secondary analyses - *Systematic reviews, literature reviews, expert consensus/ theoretical papers:* - Eligible if they propose a *new* method of discontinuation or specific recommendations for discontinuation of antipsychotics. Not eligible if article simply summarises the methods used in other studies. A “new method” is defined as a tapering strategy developed by the authors, based on clinical studies or literature included in the review. - *Clinical guidelines:* - Eligible if a method of dose reduction/ discontinuation is described, or if specific recommendations for tapering of antipsychotics are provided. | - Non-English language articles. - Ineligible study designs are: - Case studies - Research reports - Books/book chapters - Theses, conference abstracts, posters - Corrections/ Comments - Letters |
