# Supplementary material for: A Scoping Review of Methods, Guidelines, and Protocols Used for Dose Reduction or Discontinuation of Antipsychotic Medication in People Affected by Psychotic Disorders
Source: Schizophr Bull. 2025 Oct 28;52(5):sbaf181. doi: 10.1093/schbul/sbaf181 (PMC13645659; doi:10.1093/schbul/sbaf181)
Supplement: Supplementary file 4 [file supplementary_table_4_sbaf181.docx]

Supplementary Table 4. Full description of dose reduction and discontinuation methods

| **Author, year (reference)**  **(Relevant related studies)** | **Method description** | **Discontinuation/ reduction method category** |
| --- | --- | --- |
| Abrupt discontinuation / dose reduction methods (n=8) | | |
| Flint, 2019 (1)  (2, 3) | Participants were randomized under double-blind conditions to either continue olanzapine or switch from olanzapine to identically appearing placebo pills over a 4-week taper of olanzapine. The double-blind taper was conducted according to a schedule for the substitution of blinded olanzapine or placebo for open-label olanzapine, based on the number of olanzapine pills that the participant was taking at the time of randomization. | Abrupt - immediate |
| Brown, 2003 (4) | This study examined the effect of typical antipsychotic discontinuation on cocaine and amphetamine use in patients with psychiatric illnesses. The discontinuation group (n= 12) undertook abrupt discontinuation of antipsychotic medication over 2-4weeks, while the control group (n=12) continued antipsychotic medication. Specifically, the dosage of typical antipsychotics was reduced at baseline or at week 1 for those on low dosages. The atypical antipsychotic quetiapine was instituted, when necessary, for psychosis in the discontinuation group (n = 8). | Abrupt – fixed percentage/ dose |
| Rothschild & Duval, 2003 (5) | Perphenazine was reduced each week by 25% of the total dose for 4 weeks. A gradual dose reduction schedule was chosen to allow for recognition of symptoms of impending relapse and to minimize discontinuation side effects. Patients remained on treatment with their current dose of fluoxetine. For patients who exhibited impending relapse, perphenazine was immediately restarted, and the patients were titrated to their previous dose within 1 week. At the 8-month timepoint, an attempt was made to taper perphenazine for those patients who were still taking it using the same schedule that was used after 4 months of treatment. All patients were maintained on fluoxetine treatment for 12 months, then tapered off fluoxetine treatment. | Abrupt – fixed percentage/ dose |
| Kinon, 2004 (6) | During the 2-week dose reduction period, patients experienced an approximate 75% reduction in the dose that they had received immediately prior to the reduction period. Thus, patients receiving 20 mg of olanzapine during the treatment phase received 5 mg during the medication withdrawal period, while those patients receiving 5, 10 or 15 mg of olanzapine during the treatment phase received 2.5 mg during the medication withdrawal period. The dose of olanzapine could not be adjusted during the dose reduction periods. Study arm A underwent one dose reduction, while study arm B underwent two separate dose reductions; the first reduction of 75% was followed by 10-week resumption of medication, and finally a second reduction of 75%, remaining off the medication from this point. | Abrupt – fixed percentage/ dose |
| Takeuchi, 2013 (7)  (8-11) | In the reduction group, risperidone or olanzapine was reduced by 25% at baseline and week 4, followed by the treatment with half the baseline dose over the next 24 weeks. For safety reasons, the dose was not reduced beyond the lower limit of the dose range recommended for maintenance treatment of schizophrenia, i.e., 2 mg/day for risperidone and 5 mg/day for olanzapine. | Abrupt – fixed percentage/ dose |
| Graff-Guerrero, 2015 (12)  (13-17) | The antipsychotic dose was reduced by up to 40% of the baseline dosage, with a lower limit of 7.5 mg/d for olanzapine or 1.5 mg/d for risperidone based on published clinical guidelines. The dose was reduced weekly by 2.5mg for olanzapine and 0.5mg for risperidone. | Abrupt – fixed percentage/ dose |
| Ozawa, 2019 (18)  (8) | In the dose reduction group, the dose was reduced to an individually estimated target dose that corresponds to 65% dopamine D2 receptor blockade at trough. The dose was reduced to each individually estimated target dose in 4 weeks, and the participants were then treated with this target dose during a 52-week follow-up. Maintenance group received the same doses for 52 weeks. | Abrupt – fixed percentage/ dose |
| Kim, 2021 (19) | After enrollment, patients received their first [18F]DOPA PET scans (baseline) with no change in their maintenance treatment. After the first scan, antipsychotic discontinuation was completed within 4 weeks at the rate of 25% reduction in dosage per week. Other concurrently prescribed psychotropic medications at baseline were maintained as needed. Patients underwent their second PET scans, two weeks after the completion of antipsychotic discontinuation; their [11C]raclopride PET scans were conducted a week after the second [18F]DOPA PET. To ascertain the washout of antipsychotic drugs, blood samples for the measurement of antipsychotic plasma concentrations were obtained when [11C]raclopride PET scans were conducted. Patients were followed up for 12 weeks after the completion of antipsychotic discontinuation to assess their clinical symptoms and to determine if they experienced a psychotic relapse. | Abrupt – fixed percentage/ dose |
| Gradual discontinuation/ dose reduction methods (n=20) | | |
| Rouillon, 2008 (20) | Dose reduction group – the dose of olanzapine was reduced in increments of 2.5mg and the number of increments was based on response and clinical judgement. Dose reduction took up to 26 weeks (the dose was reduced once (at inclusion) for 22 patients (46%), twice for 18 patients (38%) and on 3 or 4 times for 9 patients (18%). For one patient (with 3 episodes of dose reduction) the dose was reduced once by 5 mg, this patient did not need a dose increment after. | Gradual - fixed dose |
| Gaebel, 2011 (21)  (22-24) | In the dose reduction group, the previously administered antipsychotic was discontinued in a stepwise fashion over a maximum of 3 months. For patients still receiving blinded study drug treatment, dose reduction was predefined in 1 mg steps every 1 to 2 weeks but could be adapted to patient requirements; for participants receiving open treatment, the medication dose was reduced in a stepwise fashion at the study physician's discretion. | Gradual - fixed dose |
| Bogers, 2024 (25) | In the dose reduction condition, the dose was gradually reduced to an equivalent of 5 mg of haloperidol per day. For those on an equivalent of 10mg of haloperidol, the reduction was done immediately and for those on a higher dose, it was done gradually over several steps, in increments of 5mg haloperidol equivalent, over 24 weeks. | Gradual - fixed dose |
| Lehman, 2004 (26) | Discontinuation should be gradual (e.g., reducing the dose by 10% per month). Additional precautions should be taken to minimize the risk of a psychotic relapse. Indefinite maintenance antipsychotic medication is recommended for patients who have had multiple prior episodes or two episodes within 5 years | Gradual - fixed percentage from original dose |
| Wang, 2010 (27)  (28-31) | All patients who met the study criteria were randomly assigned to a 4-week, 26-week or no-dose-reduction group. Patients in the 4-week group were maintained with the initial therapeutic risperidone dose for 4 weeks, after which the dose was reduced gradually (0.5 mg every 7–10 days, depending on the starting dose) to one-half of the therapeutic dose over the next 8 weeks, which was maintained until the end of the study. Patients in the 26-week group kept the initial therapeutic risperidone dose for 26 weeks, after which it was gradually reduced (0.5 mg every 7–10 days, depending on the starting dose) to one-half over the next 8 weeks and maintained until the end of the study. In the no-dose-reduction group, patients received the full initial therapeutic risperidone dose throughout the study. | Gradual - fixed percentage from original dose |
| Grover, 2017 (32) | The dose of the antipsychotic needs to be individualized. Stable patients who do not have positive symptoms may be candidates for reduction in doses. Doses need to be reduced gradually at the rate of about 20% every 6 months till a minimum effective dose is reached. | Gradual - fixed percentage from original dose |
| Steingard, 2018 (33) | Dose reduction of 25–30% of the initial dose at intervals no more frequent than every 3 months. The goal was not necessarily to stop the drug completely but to establish a new minimally effective dose (MED). Patients who were more cautious could reduce at a slower rate. If a patient was able to remain stable for 3–6 months after dose reduction, it was assumed a new MED was established. Decisions were made in a collaborative way and patients were free to stop or begin the taper at any time. | Gradual - fixed percentage from original dose |
| Zhou, 2018 (34) | In the dose-reduction group, the dose of risperidone or olanzapine was reduced by 25% for the first 4 weeks, then reduced by 50% of the original dose for the next 12 weeks and then maintained at this dose until the end of the study. For clinical safety, the dose was not allowed to go below 2 mg/day for risperidone and 5 mg/day for olanzapine, which is the minimum prescribed recommended dose for maintenance treatment in schizophrenia. For the dose-maintenance group, participants were prescribed the baseline dose throughout the whole study. | Gradual – fixed percentage from original dose |
| Weller, 2019 (35)  (36) | Participants randomized to gradual dose reduction group will have medication tapered by 25% (or as close as possible to 25%) of the pre-reduction dose every month for 3 months, until a clinically safe dose is reached or discontinuation. | Gradual - fixed percentage from original dose |
| Huhn, 2021 (37)  (38) | In the experimental group, antipsychotic dose was gradually reduced and stopped if possible. The initial antipsychotic dose should be reduced by 1/6 every other week for the first 3 months, but this was adapted for each patient individually. Antipsychotic doses were reduced as far as possible for the first 3 months and then patients were followed up with stable medication for 3 months. | Gradual - fixed percentage from original dose |
| Stürup, 2022 (39)  (40, 41) | Tapered discontinuation: The psychiatrist managed the tapering of antipsychotic medication, and guidelines were: (1) 25% monthly reduction of baseline dose, (2) with a minimum of five half-lives between each reduction, (3) duration of at least 6 months, and (4) with regular assessments and evaluations. | Gradual - fixed percentage from original dose |
| Wunderink, 2024 (42) | In first-episode patients, 20%–30% of the original effective dose can be safely reduced from 1 month after remission of positive symptoms and each following step every 4 weeks. In multiple-episode and chronic patients, the same method can be applied, using the same percentages of reduction of 20%–30% of the previous dose, but the intervals between the reduction steps should be much longer. If feasible and acceptable to the patient, the intervals should be more than 1 month, rather 3 up to 6 months. | Gradual - fixed percentage from original dose |
| Volavka, 2000 (43)  (44) | The experimental group experienced a gradual dose reduction over a period of 12 weeks. This strategy involved dose reduction in successive steps of 4 weeks duration, to one-half, one-fourth, and, finally, one-eight of its initial value (reduction rate 50%-25%-12.5%). Dose reductions occurred every 3 weeks for 12 weeks. The rate of dose reduction was adapted from Branchey et al., 1981 (44). | Gradual - fixed percentage from most recent dose |
| Nishikawa, 2007 (45) | There were 5 steps in the dose reduction strategy and in each step, the dose was reduced by 50% from the most recent dose. The overall time to discontinuation was 6 months but the duration of each step was determined by the treating consultant psychiatrist. | Gradual - fixed percentage from most recent dose |
| Begemann, 2020 (46)  (47) | Experimental group - Gradual discontinuation of medication according to discontinuation schedules developed by study team for common antipsychotic drugs. Duration of discontinuation depends on starting dose (average duration is 3 months to discontinuation). When psychotic symptoms occur, treatment with antipsychotic medication is restarted in the dose that patients used when the first symptomatic remission occurred. Control group - Continuation of medication for 1 year following remission, allowing for a 25% dose reduction. After 1-year, further continuation or gradual discontinuation of medication is offered to eligible patients, following the same tapering protocol above. | Gradual – variable dose |
| Moncrieff, 2023 (48)  (49-51) | An individualised reduction schedule was devised by the research team for each participant in the reduction arm, based on clinical judgement and adjusted according to the participant’s initial antipsychotic regimen. The dose was reduced incrementally every 1 or 2 months, focusing on one drug at a time where participants are taking more than one antipsychotic. The rate of reduction varies according to baseline dose, with most schedules aiming for discontinuation within 12 months, but some lasting longer where baseline doses are high. Participants were seen by the psychiatrist every 2 months for the duration of the reduction, to adjust the medication regimen and monitor mental state. The participants were offered the option to discontinue antipsychotic medication completely if the reduction progresses well, or to reduce to a very low dose, defined as the equivalent of 2 mg of haloperidol a day or less, which is lower than the minimum recommended therapeutic dose for most antipsychotics. For the reductions, there was no set amount of reduction for each step or overall, for each individual. The reduction group reduced by 67% of their baseline dose overall at some point during the 2-year trial (based on the lowest amount people got down to), but by 2 years the overall reduction was 33%. | Gradual – variable dose from original dose |
| Hui, 2022 (52) | Medications should be tapered gradually over 6–12 months and doses should be reduced gradually, at an individualized rate, and each reduction should not exceed 25% of the previous dose. Final antipsychotic dose before discontinuation should be equivalent ≤1 mg risperidone.  In the case of long-acting injectable antipsychotics, the lowest dose should be tried (25mg for paliperidone palmitate) with regular or prolonged intervals between injections. For monthly aripiprazole injections, patients should be switched to oral antipsychotics, given that the lowest dose of aripiprazole once-monthly (300 mg) is comparable with an oral dose of 15 mg. With this lowest possible dose, the recovered state should be maintained for at least 3–6 months before discontinuation. | Gradual - variable percentage from most recent dose |
| Horowitz, 2021 (53) | Hyperbolic method - antipsychotic dose reductions based on D2 receptor occupancy. Gradual reduction over months to years, with relatively larger reductions at highest doses of antipsychotic and as tapering progresses, smaller reductions at lower doses. Nine “evenly spaced” reductions based on D2 occupancy down to 0% occupancy are recommended. The D2 occupancy of the minimum effective dose is not the same for each antipsychotic, therefore, the intervals are not evenly spaced across different antipsychotics. Reduction rate should be titrated to the ability of the patient to tolerate reductions. For example, the dose could be reduced by an amount calculated to reduce 10 percentage points of the drug’s D2 blockade (approximately equal to a reduction in 25% - 50% of the most recent dose) every 3 to 6 months. However, smaller reductions, such as 10% of the most recent dose, made every month may be more tolerable to the individual. Final doses before complete cessation will need to be very small to prevent a large decrease in D2 blockade. This may need to be as small as 2.5% the original therapeutic dose. An example tapering regime for haloperidol, taking into account ease of administration and D2 occupancy, might be a reduction in dose from 4 to 3 to 2 to 1.5 to 1 to 0.75 to 0.5 to 0.375 to 0.25 to 0.125 to 0.06 to 0 mg, with tablet splitting and liquid formulations required for the lower doses. The size of reductions may be decreased or the time between reductions increased if patients experience a significant increase in symptoms.  In the case of long-acting injectable antipsychotics, 3-monthly paliperidone depot takes 52 weeks to reach a steady state and may be tapered over 3 years by yearly dose reductions equivalent to approximately 30% D2 occupancy (equivalent to reducing by 10 percentage points of D2 occupancy every 4 months), with the time taken to D2 reach a steady state providing time for neural adaptation. Very small doses of the depot would be required for final dosing (eg, 90 mg, equivalent to 30% D2 occupancy). For drugs with short half-lives like clozapine or quetiapine, more caution may be required and it may be necessary to reduce doses by 2.5–5 percentage points of D2 (or cholinergic or histaminergic) occupancy every 6–12 weeks, depending on individual responses. | Gradual – variable dose (hyperbolic) |
| Blackman, 2022 (54) | The authors recommend using the hyperbolic discontinuation regime for elective discontinuation of clozapine (adapted from Horowitz et al 2021), over a minimum of 6 months. Hyperbolic discontinuation involves dose reductions in increasingly smaller increments that approximate linear decreases in receptor occupancy. Each step is equivalent to approximating a 2.5 percentage point reduction in D2 blockade. Suggested interval between dose changes are 6−12 weeks, however this can be tailored to the individual and a slower taper can be achieved by increasing the interval between reductions and/or by smaller dose reductions. | Gradual - variable dose (hyperbolic) |
| Liu, 2023 (55)  (56-59) | The initial dose reduction was by no more than 25% of the baseline antipsychotic dose, and each step was done in 6-month intervals. The subsequent dose reduction was a reiteration of the previous step, cutting off one-quarter of the current dose, yielding 9/16 (3/4 × 3/4) of baseline dose. | Gradual - variable dose (hyperbolic) |
| Discontinuation/ dose reduction methods for LAI antipsychotic medications (n=4) | | |
| Gitlin, 2001 (60) | During the first 24 weeks of the study, patients were randomly assigned to treatment in a double-blind fashion to 12 weeks of their usual fluphenazine decanoate regimen or 12 weeks of placebo treatment. Those assigned to the initial discontinuation arm were given placebo for 12 weeks followed by 12 weeks of fluphenazine decanoate, while those in the other arm were given their usual dose of fluphenazine decanoate followed by placebo. | Abrupt - immediate |
| Khazaie & Shakeri, 2005 (61) | Patients receiving 25 mg of intramuscular fluphenazine decanoate every 2 weeks were randomly assigned to the dose reduction group and received 25 mg of fluphenazine decanoate every 6 weeks for 54 weeks. | Gradual - fixed percentage from original dose |
| Emsley, 2012 (62) | For patients taking higher than the minimum dose of Risperidone long-acting injection (25mg every 2 weeks), medication was tapered over a period of up to 6 weeks when necessary. | Gradual - fixed dose |
| Emsley, 2014 (63) | Both the experimental and control groups discontinued antipsychotics, but only the experimental group received Omega 3 and an antioxidant. In the experimental group (n=21), antipsychotic treatment was tapered and discontinued over 6 months and participants received Omega-3 fatty acids plus an antioxidant (omega 3 ω−3 PUFAs + α-LA); In the control group (n=12), antipsychotic treatment was tapered and discontinued over 6 months and participants received placebo. | Gradual – variable percentage from most recent dose |

*Abbreviations:* Long-acting injectable (LAI)

**References**

1.	Flint AJ, Meyers BS, Rothschild AJ, Whyte EM, Alexopoulos GS, Rudorfer MV, et al. Effect of continuing olanzapine vs placebo on relapse among patients with psychotic depression in remission: The STOP-PD II randomized clinical trial. JAMA. 2019;322(7):622-31.

2.	Alexopoulos G, Flint A, Rothschild A, Whyte E. Sustaining remission of psychotic depression (STOP-PD): trial registration. New York: Weill Medical College of Cornell University; 2011 [Available from: <https://clinicaltrials.gov/study/NCT01427608?term=NCT01427608&rank=1&tab=results>].

3.	Flint AJ, Meyers BS, Rothschild AJ, Whyte EM, Mulsant BH, Rudorfer MV, et al. Sustaining remission of psychotic depression: rationale, design and methodology of STOP-PD II. BMC Psychiatry. 2013;13:38.

4.	Brown ES, Nejtek VA, Perantie DC, Rajan Thomas N, Rush AJ. Cocaine and amphetamine use in patients with psychiatric illness: a randomized trial of typical antipsychotic continuation or discontinuation. J Clin Psychopharmacol. 2003;23(4):384-8.

5.	Rothschild AJ, Duval SE. How long should patients with psychotic depression stay on the antipsychotic medication? J Clin Psychiatry. 2003;64(4):390-6.

6.	Kinon BJ, Jeste DV, Kollack-Walker S, Stauffer V, Liu-Seifert H. Olanzapine treatment for tardive dyskinesia in schizophrenia patients: a prospective clinical trial with patients randomized to blinded dose reduction periods. Prog Neuropsychopharmacol Biol Psychiatry. 2004;28(6):985-96.

7.	Takeuchi H, Suzuki T, Remington G, Bies RR, Abe T, Graff-Guerrero A, et al. Effects of risperidone and olanzapine dose reduction on cognitive function in stable patients with schizophrenia: an open-label, randomized, controlled, pilot study. Schizophr Bull. 2013;39(5):993-8.

8.	Uchida H. Effects of antipsychotic reduction on negative subjective experience and cognitive function: trial registration. Tokyo, Japan: Keio University, School of Medicine; 2009 [Available from: <https://center6.umin.ac.jp/cgi-open-bin/ctr_e/ctr_view.cgi?recptno=R000002200>].

9.	Takeuchi H, Suzuki T, Bies RR, Remington G, Watanabe K, Mimura M, et al. Dose reduction of risperidone and olanzapine and estimated dopamine D₂ receptor occupancy in stable patients with schizophrenia: findings from an open-label, randomized, controlled study. J Clin Psychiatry. 2014;75(11):1209-14.

10.	Takeuchi H, Suzuki T, Remington G, Watanabe K, Mimura M, Uchida H. Lack of effect of risperidone or olanzapine dose reduction on subjective experiences in stable patients with schizophrenia. Psychiatry Res. 2014;218(1-2):244-6.

11.	Gerretsen P, Takeuchi H, Ozzoude M, Graff-Guerrero A, Uchida H. Insight into illness and its relationship to illness severity, cognition and estimated antipsychotic dopamine receptor occupancy in schizophrenia: An antipsychotic dose reduction study. Psychiatry Res. 2017;251:20-5.

12.	Graff-Guerrero A, Rajji TK, Mulsant BH, Nakajima S, Caravaggio F, Suzuki T, et al. Evaluation of antipsychotic dose reduction in late-life schizophrenia: a prospective dopamine D2/3 receptor occupancy study. JAMA Psychiatry. 2015;72(9):927-34.

13.	Uchida H, Suzuki T, Graff-Guerrero A, Mulsant BH, Pollock BG, Arenovich T, et al. Therapeutic window for striatal dopamine D(2/3) receptor occupancy in older patients with schizophrenia: a pilot PET study. Am J Geriatr Psychiatry. 2014;22(10):1007-16.

14.	Nakajima S, Uchida H, Bies RR, Caravaggio F, Suzuki T, Plitman E, et al. Dopamine D2/3 receptor occupancy following dose reduction is predictable with minimal plasma antipsychotic concentrations: an open-label clinical trial. Schizophr Bull. 2016;42(1):212-9.

15.	Iwata Y, Nakajima S, Caravaggio F, Suzuki T, Uchida H, Plitman E, et al. Threshold of dopamine D2/3 receptor occupancy for hyperprolactinemia in older patients with schizophrenia. J Clin Psychiatry. 2016;77(12):e1557-e63.

16.	Fervaha G, Caravaggio F, Mamo DC, Mulsant BH, Pollock BG, Nakajima S, et al. Lack of association between dopaminergic antagonism and negative symptoms in schizophrenia: a positron emission tomography dopamine D2/3 receptor occupancy study. Psychopharmacology (Berl). 2016;233(21-22):3803-13.

17.	Rajji TK, Mulsant BH, Nakajima S, Caravaggio F, Suzuki T, Uchida H, et al. Cognition and dopamine D(2) receptor availability in the striatum in older patients with schizophrenia. Am J Geriatr Psychiatry. 2017;25(1):1-10.

18.	Ozawa C, Bies RR, Pillai N, Suzuki T, Mimura M, Uchida H. Model-guided antipsychotic dose reduction in schizophrenia: a pilot, single-blind randomized controlled trial. J Clin Psychopharmacol. 2019;39(4):329-35.

19.	Kim S, Shin SH, Santangelo B, Veronese M, Kang SK, Lee JS, et al. Dopamine dysregulation in psychotic relapse after antipsychotic discontinuation: an [(18)F]DOPA and [(11)C]raclopride PET study in first-episode psychosis. Mol Psychiatry. 2021;26(7):3476-88.

20.	Rouillon F, Chartier F, Gasquet I. Strategies of treatment with olanzapine in schizophrenic patients during stable phase: results of a pilot study. European neuropsychopharmacology. 2008;18(9):646‐52.

21.	Gaebel W, Riesbeck M, Wölwer W, Klimke A, Eickhoff M, von Wilmsdorff M, et al. Relapse prevention in first-episode schizophrenia-maintenance vs intermittent drug treatment with prodrome-based early intervention: results of a randomized controlled trial within the German Research Network on Schizophrenia. J Clin Psychiatry. 2011;72(2):205-18.

22.	Gaebel W. Maintenance treatment vs. stepwise drug discontinuation in first-episode schizophrenia: trial registration. Duesseldorf: Heinrich-Heine University; 2005 [Available from: <https://clinicaltrials.gov/study/NCT00159120>].

23.	Gaebel W, Möller HJ, Buchkremer G, Ohmann C, Riesbeck M, Wölwer W, et al. Pharmacological long-term treatment strategies in first episode schizophrenia--study design and preliminary results of an ongoing RCT within the German Research Network on Schizophrenia. Eur Arch Psychiatry Clin Neurosci. 2004;254(2):129-40.

24.	Gaebel W, Riesbeck M, Wölwer W, Klimke A, Eickhoff M, von Wilmsdorff M, et al. Predictors for symptom re-exacerbation after targeted stepwise drug discontinuation in first-episode schizophrenia: results of the first-episode study within the German research network on schizophrenia. Schizophrenia Research. 2016;170(1):168-76.

25.	Bogers JPAM, Blömer JA, de Haan L. Cognitive effects of reducing first-generation antipsychotic dose compared to switching to ziprasidone in long-stay patients with schizophrenia. Journal of Clinical Medicine. 2024;13(7).

26.	Lehman AF, Lieberman J, Dixon L, McGlashan T, Miller A, Perkins D, et al. Practice guideline for the treatment of patients with schizophrenia. 2004.

27.	Wang CY, Xiang YT, Cai ZJ, Weng YZ, Bo QJ, Zhao JP, et al. Risperidone maintenance treatment in schizophrenia: a randomized, controlled trial. Am J Psychiatry. 2010;167(6):676-85.

28.	Xiang YT, Wang CY, Ungvari GS, Kreyenbuhl JA, Chiu HF, Lai KY, et al. Weight changes and their associations with demographic and clinical characteristics in risperidone maintenance treatment for schizophrenia. Pharmacopsychiatry. 2011;44(4):135-41.

29.	Xiang YT, Wang CY, Weng YZ, Bo QJ, Chiu HF, Chan SS, et al. Predictors of relapse in Chinese schizophrenia patients: a prospective, multi-center study. Soc Psychiatry Psychiatr Epidemiol. 2011;46(12):1325-30.

30.	Bo QJ, Li XB, Wang ZM, Li AN, Ma X, Wang CY. Extrapyramidal symptoms during risperidone maintenance treatment in schizophrenia: a prospective, multicenter study. J Clin Psychopharmacol. 2016;36(2):125-9.

31.	Bo Q, Li F, Li X, Wang Z, Dong F, He F, et al. Symptomatic remission in schizophrenia: results from a risperidone maintenance treatment study. Psychiatry Res. 2017;258:289-94.

32.	Grover S, Chakrabarti S, Kulhara P, Avasthi A. Clinical practice guidelines for management of schizophrenia. Indian J Psychiatry. 2017;59(Suppl 1):S19-S33.

33.	Steingard S. Five year outcomes of tapering antipsychotic drug doses in a community mental health center. Community Ment Health J. 2018;54(8):1097-100.

34.	Zhou Y, Li G, Li D, Cui H, Ning Y. Dose reduction of risperidone and olanzapine can improve cognitive function and negative symptoms in stable schizophrenic patients: A single-blinded, 52-week, randomized controlled study. J Psychopharmacol. 2018;32(5):524-32.

35.	Weller A, Gleeson J, Alvarez-Jimenez M, McGorry P, Nelson B, Allott K, et al. Can antipsychotic dose reduction lead to better functional recovery in first-episode psychosis? A randomized controlled-trial of antipsychotic dose reduction. The reduce trial: Study protocol. Early Interv Psychiatry. 2019;13(6):1345-56.

36.	Killackey E. Does antipsychotic dose reduction in combination with Evidence-Based Intensive Recovery Treatment (EBIRT) lead to better functional recovery in first episode psychosis: a randomised controlled trial: trial registration. Victoria, Australia: Orygen, the National Centre of Excellence in Youth Mental Health; 2017 [Available from: <https://www.anzctr.org.au/Trial/Registration/TrialReview.aspx?ACTRN=ACTRN12617000870358>].

37.	Huhn M, Leucht C, Rothe P, Dold M, Heres S, Bornschein S, et al. Reducing antipsychotic drugs in stable patients with chronic schizophrenia or schizoaffective disorder: a randomized controlled pilot trial. Eur Arch Psychiatry Clin Neurosci. 2021;271(2):293-302.

38.	Leucht S, Dold M. Evaluation of the necessity of long-term pharmacological treatment with antipsychotics in schizophrenic patients: trial registration. Munich: Technical University of Munich; 2015 [Available from: <https://clinicaltrials.gov/study/NCT02307396?term=NCT02307396&rank=1>].

39.	Stürup AE, Hjorthøj C, Albert N, Dolmer S, Birk M, Ebdrup BH, et al. Tapered discontinuation vs. maintenance therapy of antipsychotic medication in patients with first-episode schizophrenia: Obstacles, findings, and lessons learned in the terminated randomized clinical trial TAILOR. Front Psychiatry. 2022;13:910703.

40.	Nordentoft M. TAILOR - a randomized clinical trial: Tapered discontinuation versus maintenance therapy of antipsychotic medication in patients with newly diagnosed schizophrenia or schizophreniform psychosis in remission of psychotic symptoms: Trial registration. Denmark: Trygfonden; 2016 [Available from: <https://www.clinicaltrialsregister.eu/ctr-search/trial/2016-000565-23/DK#B>].

41.	Stürup AE, Jensen HD, Dolmer S, Birk M, Albert N, Nielsen M, et al. TAILOR – tapered discontinuation versus maintenance therapy of antipsychotic medication in patients with newly diagnosed schizophrenia or persistent delusional disorder in remission of psychotic symptoms: study protocol for a randomized clinical trial. Trials. 2017;18(1):445.

42.	Wunderink L. Changing vistas of psychosis and antipsychotic drug dosing toward personalized management of antipsychotics in clinical practice. Psychiatr Rehabil J. 2024.

43.	Volavka J, Cooper TB, Czobor P, Lindenmayer JP, Citrome LL, Mohr P, et al. High-dose treatment with haloperidol: the effect of dose reduction. J Clin Psychopharmacol. 2000;20(2):252-6.

44.	Branchey MH, Branchey LB, Richardson MA. Effects of neuroleptic adjustment on clinical condition and tardive dyskinesia in schizophrenic patients. Am J Psychiatry. 1981;138(5):608-12.

45.	Nishikawa T, Hayashi T, Koga I, Uchida Y. Neuroleptic withdrawal with remitted schizophrenics: a naturalistic follow-up study. Psychiatry. 2007;70(1):68-79.

46.	Begemann MJH, Thompson IA, Veling W, Gangadin SS, Geraets CNW, van 't Hag E, et al. To continue or not to continue? Antipsychotic medication maintenance versus dose-reduction/discontinuation in first episode psychosis: HAMLETT, a pragmatic multicenter single-blind randomized controlled trial. Trials. 2020;21(1):147.

47.	Begemann MJH. HAMLETT: Handling Antipsychotic Medication: Long-term Evaluation of Targeted Treatment, A pragmatic single-blind randomised controlled trial of continuation versus discontinuation/ dose reduction of antipsychotic medication in patients remitted after a first episode of psychosis: Trial registration. Netherlands: University Medical Center Groningen; 2017 [Available from: <https://onderzoekmetmensen.nl/en/trial/55504>].

48.	Moncrieff J, Crellin N, Stansfeld J, Cooper R, Marston L, Freemantle N, et al. Antipsychotic dose reduction and discontinuation versus maintenance treatment in people with schizophrenia and other recurrent psychotic disorders in England (the RADAR trial): an open, parallel-group, randomised controlled trial. The Lancet Psychiatry. 2023;10(11):848-59.

49.	Moncrieff J. Research into antipsychotic discontinuation and reduction trial: trial registration. London: University College London; 2016 [Available from: <https://clinicaltrials.gov/study/NCT03559426?term=NCT03559426&rank=1>].

50.	Moncrieff J, Lewis G, Freemantle N, Johnson S, Barnes TRE, Morant N, et al. Randomised controlled trial of gradual antipsychotic reduction and discontinuation in people with schizophrenia and related disorders: the RADAR trial (Research into Antipsychotic Discontinuation and Reduction). BMJ Open. 2019;9(11):e030912.

51.	Bray G, Moncrieff J, Priebe S, Marston L, Lewis G, Haynes N, et al. Cost-utility analysis of antipsychotic reduction and discontinuation in patients with long-term schizophrenia and psychosis in english mental health trusts: The RADAR study. Value Health. 2025;28(3):358-67.

52.	Hui C, Chen E, Swapna V, Tagata H, Mizuno M, Liu CC, et al. Guidelines for discontinuation of antipsychotics in patients who recover from first-episode schizophrenia spectrum disorders: derived from the aggregated opinions of Asian Network of Early Psychosis experts and literature review. Int J Neuropsychopharmacol. 2022;25(9):737-58.

53.	Horowitz MA, Jauhar S, Natesan S, Murray RM, Taylor D. A method for tapering antipsychotic treatment that may minimize the risk of relapse. Schizophrenia Bulletin. 2021;47(4):1116-29.

54.	Blackman G, Oloyede E, Horowitz M, Harland R, Taylor D, MacCabe J, et al. Reducing the risk of withdrawal symptoms and relapse following clozapine discontinuation - is it feasible to develop evidence-based guidelines? Schizophr Bull. 2022;48(1):176-89.

55.	Liu CC, Hsieh MH, Chien YL, Liu CM, Lin YT, Hwang TJ, et al. Guided antipsychotic reduction to reach minimum effective dose (GARMED) in patients with remitted psychosis: a 2-year randomized controlled trial with a naturalistic cohort. Psychol Med. 2023;53(15):7078-86.

56.	Liu CC. Guided dose reduction of antipsychotic in patients with psychosis in remitted states (GDR): Trial registration. Taiwan: National Taiwan University Hospital; 2017 [Available from: <https://clinicaltrials.gov/study/NCT03248180?term=NCT03248180&rank=1>].

57.	Liu C-C, Takeuchi H. Achieving the lowest effective antipsychotic dose for patients with remitted psychosis: a proposed guided dose-reduction algorithm. CNS Drugs. 2019;34.

58.	Liu CC, Hsieh MH, Chien YL, Liu CM, Lin YT, Hwang TJ, et al. Protocol of guided antipsychotic reduction to reach minimum effective dose (GARMED) in patients with remitted psychosis based on pragmatic design. Early Interv Psychiatry. 2022;16(2):178-85.

59.	Liu CC, Hsieh MH, Chien YL, Liu CM, Lin YT, Hwang TJ, et al. Dose-tapering trajectories in patients with remitted psychosis undergoing guided antipsychotic reduction to reach minimum effective dose. Eur Psychiatry. 2023;66(1):e66.

60.	Gitlin M, Nuechterlein K, Subotnik KL, Ventura J, Mintz J, Fogelson DL, et al. Clinical outcome following neuroleptic discontinuation in patients with remitted recent-onset schizophrenia. Am J Psychiatry. 2001;158(11):1835-42.

61.	Khazaie H, Shakeri J. Comparative efficacy of every 2 weeks versus every 6 weeks injections of fluphenazine decanoate. Archives of Iranian Medicine. 2005;8(2):109-14.

62.	Emsley R, Oosthuizen PP, Koen L, Niehaus DJ, Martinez G. Symptom recurrence following intermittent treatment in first-episode schizophrenia successfully treated for 2 years: a 3-year open-label clinical study. J Clin Psychiatry. 2012;73(4):e541-7.

63.	Emsley R, Chiliza B, Asmal L, du Plessis S, Phahladira L, van Niekerk E, et al. A randomized, controlled trial of omega-3 fatty acids plus an antioxidant for relapse prevention after antipsychotic discontinuation in first-episode schizophrenia. Schizophr Res. 2014;158(1-3):230-5.
